# Production of the biocommodities butanol and acetone from methanol with fluorescent FAST-tagged proteins using metabolically engineered strains of *Eubacterium limosum*

**DOI:** 10.1186/s13068-021-01966-2

**Published:** 2021-05-10

**Authors:** Maximilian Flaiz, Gideon Ludwig, Frank R. Bengelsdorf, Peter Dürre

**Affiliations:** Institute of Microbiology and Biotechnology, Ulm University, Albert-Einstein-Allee 11, 89081 Ulm, Germany

**Keywords:** Acetogens, Anaerobes, C1-substrates, Fluorescence-activating and absorption shifting tag, Fluorescence reporter system, Fusion protein

## Abstract

**Background:**

The interest in using methanol as a substrate to cultivate acetogens increased in recent years since it can be sustainably produced from syngas and has the additional benefit of reducing greenhouse gas emissions. *Eubacterium limosum* is one of the few acetogens that can utilize methanol, is genetically accessible and, therefore, a promising candidate for the recombinant production of biocommodities from this C1 carbon source. Although several genetic tools are already available for certain acetogens including *E. limosum,* the use of brightly fluorescent reporter proteins is still limited.

**Results:**

In this study, we expanded the genetic toolbox of *E. limosum* by implementing the fluorescence-activating and absorption shifting tag (FAST) as a fluorescent reporter protein. Recombinant *E. limosum* strains that expressed the gene encoding FAST in an inducible and constitutive manner were constructed. Cultivation of these recombinant strains resulted in brightly fluorescent cells even under anaerobic conditions. Moreover, we produced the biocommodities butanol and acetone from methanol with recombinant *E. limosum* strains. Therefore, we used *E.* *limosum* cultures that produced FAST-tagged fusion proteins of the bifunctional acetaldehyde/alcohol dehydrogenase or the acetoacetate decarboxylase, respectively, and determined the fluorescence intensity and product concentrations during growth.

**Conclusions:**

The addition of FAST as an oxygen-independent fluorescent reporter protein expands the genetic toolbox of *E. limosum*. Moreover, our results show that FAST-tagged fusion proteins can be constructed without negatively impacting the stability, functionality, and productivity of the resulting enzyme. Finally, butanol and acetone can be produced from methanol using recombinant *E.* *limosum* strains expressing genes encoding fluorescent FAST-tagged fusion proteins.

**Supplementary Information:**

The online version contains supplementary material available at 10.1186/s13068-021-01966-2.

## Background

Acetogens are promising biocatalysts for the sustainable production of biocommodities since their ability to use carbon dioxide (CO_2_)- and carbon monoxide (CO)-containing industrial waste gases as feedstock contributes to the reduction of greenhouse gas emissions. Nevertheless, the fermentation of such synthesis gas (syngas) faces technical challenges as the mass transfer of gases into the liquid state of media is quite poor and consequently limits microbial productivity [1]. An alternative to the C1 gases as a substrate is the use of methanol, since it bears the advantage of easy transport, storage, and is completely soluble in water, and does, therefore, not suffer from mass transfer issues [2]. Methanol can be produced from CO-, CO_2_-, and H_2_-containing syngas [3] and is a cheap, sustainable, and relatively pure feedstock for a variety of bacteria [4–7]. Several acetogens can utilize methanol via the Wood–Ljungdahl pathway [8], which has been elucidated in detail for *Acetobacterium woodii* [9]. Since the production of chemicals with methanol-utilizing acetogens seems to be auspicious, it is also of great interest to expand their molecular toolbox. Molecular tools with different levels of possibilities for metabolic engineering are available for acetogens such as *A. woodii*, *Clostridium ljungdahlii*, *C. autoethanogenum*, and *E. limosum.* That includes, e.g., genomic editing tools [10–14] and the expression of recombinant pathways to produce biocommodities such as butanol [15, 16], acetone [17–19], isopropanol [20], 3-hydroxybutyrate [21], or poly(3-hydroxybutyrate) [22]. However, fluorescent reporter systems which are well-established and often used tools in molecular biology to study gene expression [23, 24], promoter activities [25, 26], or the dynamics in microbial populations and co-cultures [27–29] are still restricted for acetogens, basically due to the lack of proteins that show bright fluorescence under anaerobic conditions. Recently, the fluorescence-activating and absorption shifting tag (FAST) [30] was established in *C. acetobutylicum* [31] and *C. ljungdahlii* [32] and opened the door for application in other anaerobic bacteria, since its fluorescence is bright and independent of oxygen. FAST is a small-sized protein with a mass of 14 kDa that only shows fluorescence when it forms a non-covalent reversible complex with a fluorogenic ligand, a so-called fluorogen [30, 33]. Those fluorogens are hydroxybenzylidene rhodanine derivatives that are non-fluorescent by themselves and only show fluorescence when bound to FAST [30]. Due to the small size, FAST is perfectly suited for genetic fusion to any protein of interest (POI) [30]. Such fusion proteins enable investigations of protein localization and intracellular dynamics [31, 34–36].

**Fig. 1:**
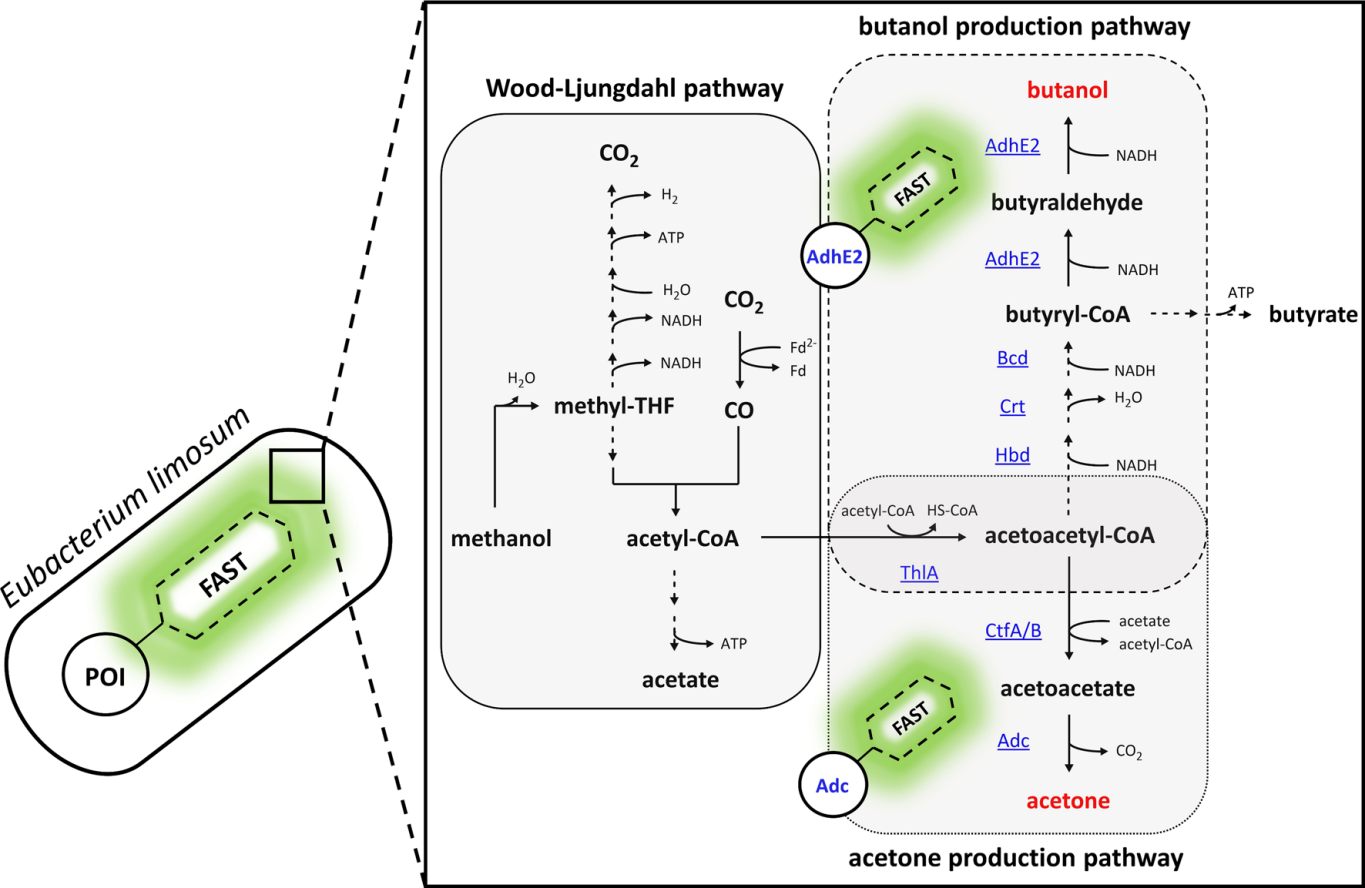
Schematic overview of the WLP based on Kremp *et al*. [9] coupled with the recombinant butanol and acetone production pathway in *E. limosum*. Butanol and acetone production can be achieved with FAST-tagged AdhE2 and Adc fusion proteins, respectively. ThlA, thiolase; CtfA/B, acetoacetyl-CoA:acetate/butyrate-CoA transferase; Adc, acetoacetate decarboxylase; Hbd, 3-hydroxybutyryl-CoA dehydrogenase; Crt, crotonase; Bcd, butyryl-CoA dehydrogenase; AdhE2, bifunctional acetaldehyde/alcohol dehydrogenase

In this study, we aimed to expand the genetic toolbox of the Gram-positive, methanol-utilizing acetogen *E. limosum* [37], which is one of the few acetogens that is genetically accessible [13, 38], by establishing FAST as a fluorescent reporter system. We used FAST to construct FAST-tagged fusion proteins. As an initial approach, the bifunctional acetaldehyde/alcohol dehydrogenase (AdhE2) from *C. acetobutylicum*, which mediates the reaction from butyryl-CoA to butanol [39], was tagged with FAST and the respective gene expressed in recombinant *E. limosum* strains (Fig. 1). Since *E.* *limosum* produces butyryl-CoA naturally [40], the heterologous expression of the gene encoding the FAST-tagged AdhE2 fusion protein is sufficient to produce butanol. As a second approach, the acetoacetate decarboxylase (Adc) originating from *C. acetobutylicum* was FAST-tagged and the respective gene used to assemble an artificial acetone production operon (APO) also including the genes *thlA* and *ctfA/B* (encoding thiolase and acetoacetyl-CoA: acetate/butyrate-CoA transferase) originating from *C. acetobutylicum* [41, 42]. Thus, acetone production can be achieved by expression of the APO using recombinant *E. limosum* strains (Fig. 1). Evidence for heterologous production of the FAST-tagged AdhE2 and Adc fusion proteins within *E.* *limosum* mutants was provided by the determination of the fluorescence intensity of the respective bacterial populations producing the FAST-tagged fusion proteins during growth. Moreover, a comparison of production patterns of the biocommodities butanol and acetone using the C1 carbon source methanol provides first insight regarding stability, functionality, and productivity of the AdhE2 and Adc FAST-tagged fusion proteins.

## Results

### Growth of *E. limosum* using different carbon sources

**Fig. 2:**
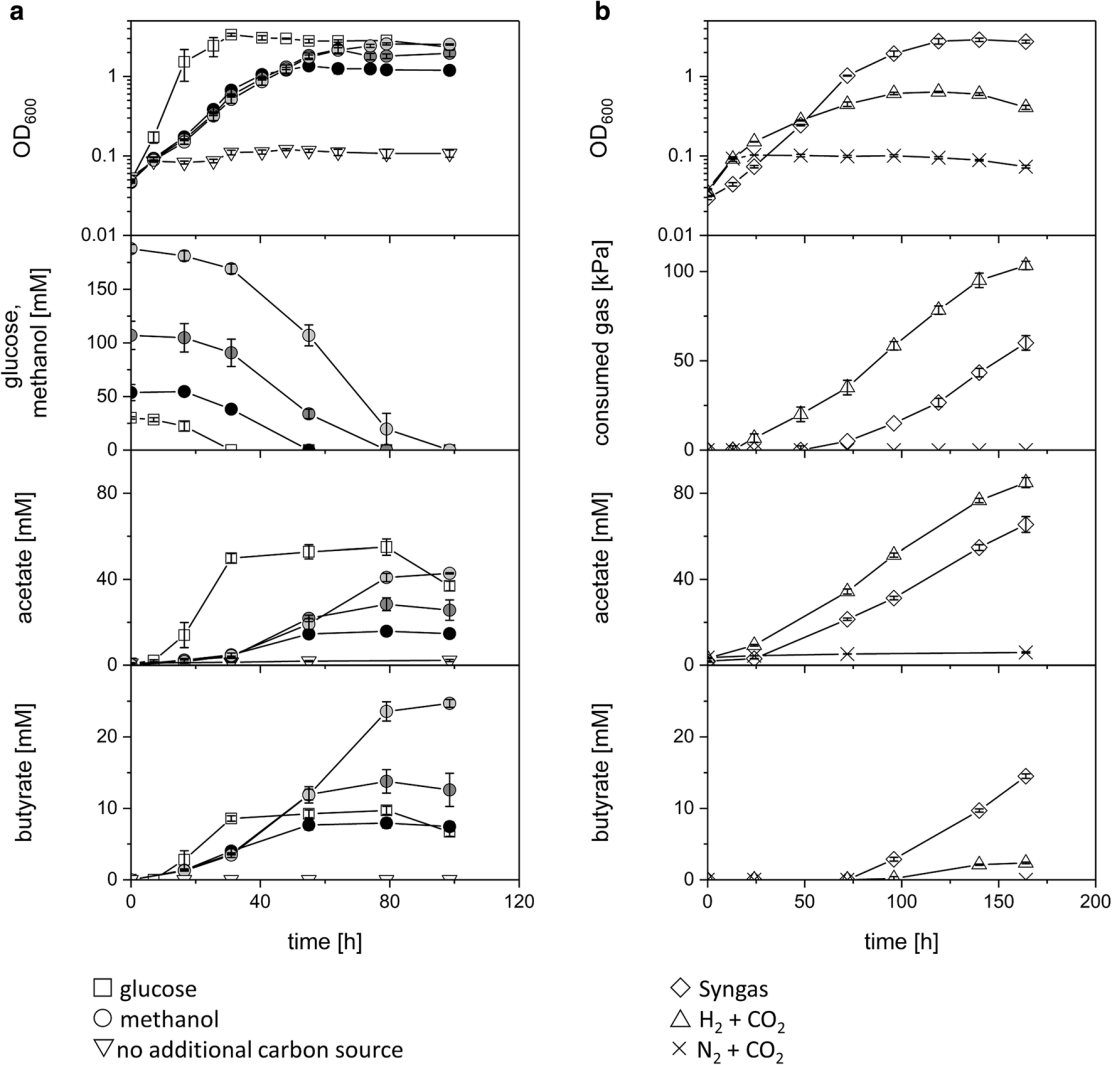
**a** Heterotrophic and **b** autotrophic growth experiments using *E. limosum* NG-6894 cultivated on different carbon and energy sources. *E. limosum* was cultivated with 30 mM glucose (white); 50 (black), 100 (dark gray), or 200 mM (gray) methanol; without additional carbon source; Syngas; H_2_ + CO_2_; N_2_ + CO_2_. OD_600_, glucose, methanol, and gas consumption (absolute value of accumulated pressure loss) as well as acetate and butyrate production were monitored. Error bars indicate standard deviations. n = 3

*E. limosum* NG-6894 was cultivated heterotrophically using glucose or methanol as well as autotrophically using H_2_ + CO_2_ and syngas as carbon and energy source. Heterotrophically cultivated cells using glucose reached a maximal optical density (OD_600_) of 2.83 with a growth rate of 0.23 h^−1^. Cell cultures grown on various methanol concentrations of 50, 100, and 200 mM showed maximal OD_600_ values of 1.4, 2, and 2.6, respectively, while having a similar growth rate of about 0.09 h^−1^ (Fig. 2a and Table 1). Autotrophically grown cells using H_2_ + CO_2_ showed the lowest growth rate of 0.02 h^−1^ and a maximal OD_600_ value of 0.6. Cultivation using syngas resulted in a maximal OD_600_ of 2.9 and a growth rate of 0.06 h^−1^ (Fig. 2b and Table 1). *E. limosum* NG-6894 produced acetate and butyrate under heterotrophic as well as under autotrophic growth conditions, while the ratio between these products varied depending on the substrates used. Acetate and butyrate production started at the end of the exponential growth phase and reached their maximum in the stationary growth phase after the substrate was consumed completely. Heterotrophically grown cells using glucose showed a product ratio of acetate:butyrate of 6:1, which shifted to 2:1 when cells were cultivated with methanol, independent of the used amount of methanol (Table 1). *E. limosum* NG-6894 cells cultivated autotrophically using H_2_ + CO_2_ produced the highest amounts of acetate (85 mM) and only low amounts of butyrate (2.4 mM), which resulted in a product ratio of 36:1. Cells grown using syngas achieved an acetate:butyrate ratio of 9:2 (Table 1). As a control, cells cultivated without the addition of a defined carbon source except for the standard medium components carbonate and yeast extract accomplished only one doubling and produced traces of acetate (2.2–6 mM).

**Table 1:** Comparison of growth characteristics and products of *E. limosum* NG-6894 cultivated using various carbon and energy sources

Substrate	Max. OD_600_	Growth rate (h^−1^)	Doubling time (h)	Products (mM)		Products (mol/100 mol substrate)		Product ratio (acetate:butyrate)^a^
				Acetate	Butyrate	Acetate	Butyrate	
Glucose								
30 mM	2.8	0.23	3	55	9.7	183.2	32.4	6:1
Methanol								
50 mM	1.4	0.09	7.7	15.8	8	29.5	14.8	2:1
100 mM	2	0.09	7.7	28.4	13.8	26.6	12.9	2:1
200 mM	2.6	0.09	7.7	42.8	24.7	22.8	13.1	2:1
Gases								
H_2_ + CO_2_	0.6	0.02	34.7	85	2.4	n.d.	n.d.	36:1
Syngas	2.9	0.06	11.6	65.5	14.5	n.d.	n.d.	9:2

^a^Product ratios were calculated for products in (mM) and (mol/100 mol substrate)

*n.d.* not determined

### Engineering of the fluorescence activation and absorption shifting tag in *E. limosum*

**Fig. 3:**
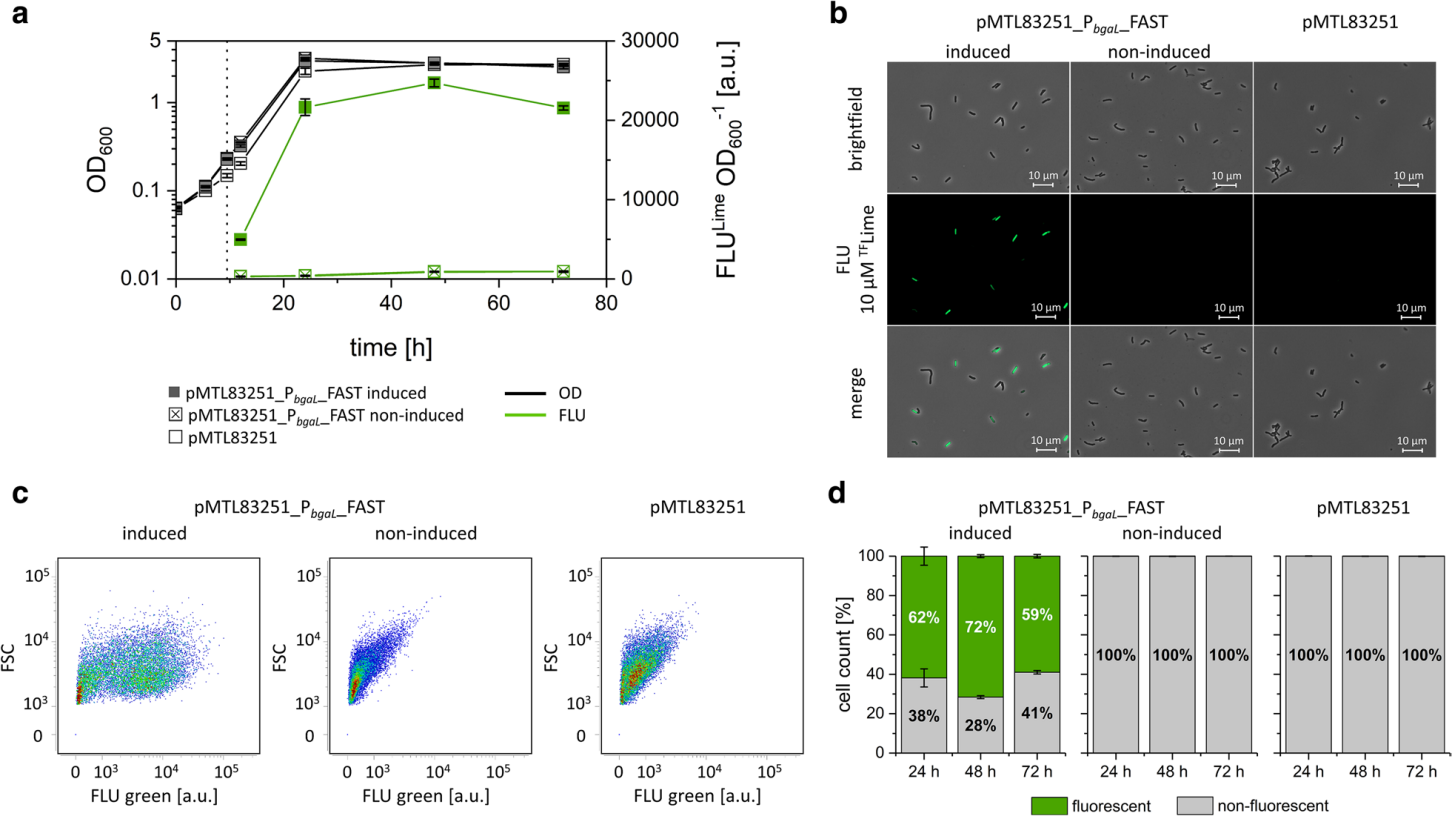
**a** Fluorescence intensity of recombinant *E. limosum* strains during growth in presence of the fluorogen ^TF^Lime. Cells were cultivated using 30 mM glucose. Monitored were OD_600_ and fluorescence intensity of *E. limosum* [pMTL83251_P_*bgaL*__FAST] with induced and non-induced gene expression as well as *E. limosum* [pMTL83251]. **b** Micrographs of *E. limosum* [pMTL83251_P_*bgaL*__FAST] and *E. limosum* [pMTL83251] after 72 h of incubation. Gene expression of cells was either induced or non-induced. **c** Density plots of *E. limosum* [pMTL83251_P_*bgaL*__FAST] and *E. limosum* [pMTL83251] after 72 h of incubation. Gene expression of cells was either induced or non-induced. **d** Number of fluorescent *E. limosum* cells determined after 24 h, 48 h, and 72 h cultivation. n = 3

After transformation, the recombinant *E. limosum* [pMTL83251_P_*bgaL*__FAST] expressed the FAST-encoding gene (*feg*) controlled by the lactose-inducible *bgaR*-P_*bgaL*_ promoter. Cells of that strain showed bright fluorescence during growth only in the presence of the fluorogen ^TF^Lime after induction of gene expression (Fig. 3). *E. limosum* [pMTL83251_P_*bgaL*__FAST] was cultivated on glucose and reached a 26-fold higher maximum fluorescence intensity after 48 h of cultivation compared to the non-induced strain. Cells of the non-induced strain did not show any fluorescence except autofluorescence in the presence of ^TF^Lime. Both, the induced and the non-induced strain reached similar maximal OD_600_ values of 3 and 3.1 after 24 h of cultivation, respectively. The empty vector control *E. limosum* [pMTL83251] reached an OD_600_ of 2.7 and showed only autofluorescence when supplemented with ^TF^Lime (Fig. 3a). Fluorescence microscopy was used to image fluorescent cells (Fig. 3b). The resulting micrographs show brightly fluorescent cells of *E. limosum* [pMTL83251_P_*bgaL*__FAST] with induced gene expression. Non-induced as well as cells of the empty vector control *E. limosum* [pMTL83251] did not show any fluorescence. Nevertheless, fluorescence microscopy revealed an inhomogeneous population of fluorescent and non-fluorescent *E. limosum* [pMTL83251_P_*bgaL*__FAST] cells. *E. limosum* [pMTL83251_P_*bgaL*__FAST] cells expressing *feg* and cultivated under equal growth conditions were analyzed at single cell level using flow cytometry (Fig. 3c). Initial autofluorescence of cells of the empty vector control strain *E. limosum* [pMTL83251] was determined and gated as non-fluorescent events. Cells of the strain *E. limosum* [pMTL83251_P_*bgaL*__FAST] with lactose-induced *feg* expression resulted in a clear shift showing a population with green fluorescence and confirming the results obtained by fluorescence microscopy. Flow cytometry data revealed that in the late exponential growth phase 62% of induced *E. limosum* [pMTL83251_P_*bgaL*__FAST] cells were fluorescent, while 38% of them were non-fluorescent. The number of fluorescent cells increased in the stationary growth phase up to 72%. In the late stationary growth phase, after 72 h of cultivation, the number of fluorescent cells decreased to 59%. The population of the non-induced cells showed no shift and were non-fluorescent during all determined time points (Fig. 3d).

**Fig. 4:**
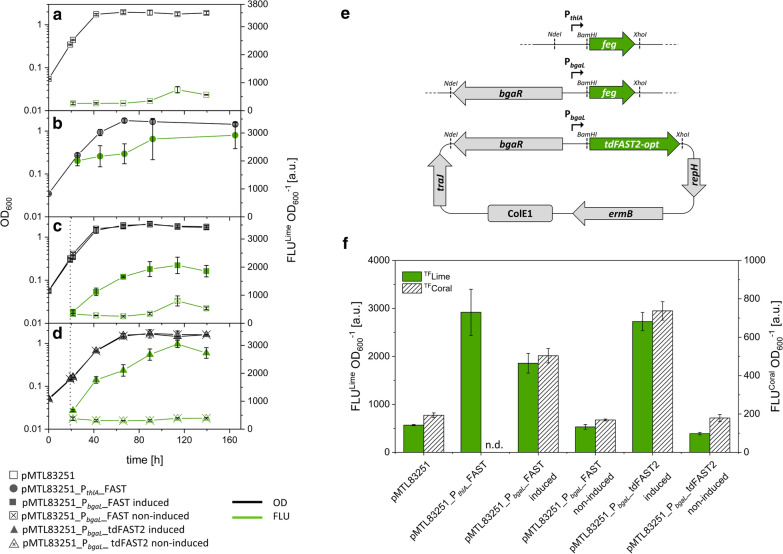
Fluorescence intensity of recombinant *E. limosum* strains grown on methanol (100 mM) in presence of the fluorogen ^TF^Lime. **a** OD_600_ and fluorescence intensity of *E. limosum* [pMTL83251], **b** *E. limosum* [pMTL83251_P_*thlA*__FAST], **c** *E. limosum* [pMTL83251_P_*bgaL*__FAST], and **d** *E. limosum* [pMTL83251_P_*bgaL*__tdFAST2] were monitored. Gene expression of *E. limosum* [pMTL83251_P_*bgaL*__FAST] and *E. limosum* [pMTL83251_P_*bgaL*__tdFAST2] was either induced by lactose or non-induced. Time of induction with lactose is indicated with the vertical dotted line. n=3, error bars show standard deviation. **e** Genetic maps of plasmids representing pMTL83251_P_*thlA*__FAST (top), pMTL83251_P_*bgaL*__FAST (middle), and pMTL83251_P_*bgaL*__tdFAST2 (bottom). Fluorescence during growth of cells harboring respective plasmids are shown in panel b, c, and d, respectively. **f** Maximal fluorescence intensity of recombinant *E. limosum* strains in the presence of the fluorogen ^TF^Lime or ^TF^Coral. Mean fluorescence was determined during stationary growth phase at an OD_600_ of ~ 1.5, n = 3, error bars show standard deviation. n = 3

Furthermore, the fluorescence of FAST-producing cells cultivated with methanol as carbon and energy source was examined. In addition to *E.* *limosum* [pMTL83251_P_*bgaL*__FAST] a strain termed *E. limosum* [pMTL83251_P_*thlA*__FAST] was constructed that expressed *feg* under the control of the constitutive P_*thlA*_ promoter. *E.* *limosum* [pMTL83251] harboring the empty vector control showed only autofluorescence when the fluorogen ^TF^Lime was supplemented (Fig. 4a). The constitutive expression of *feg* by *E. limosum* [pMTL83251_P_*thlA*__FAST] caused a clear and increasing fluorescence during growth and a maximum intensity during the stationary stage (Fig. 4b). The fluorescence intensity of cells that produced FAST constitutively was 5.1-fold higher compared to the autofluorescence of *E. limosum* [pMTL83251]. The lactose-induced expression of *feg* by *E. limosum* [pMTL83251_P_*bgaL*__FAST] caused clear fluorescence during cultivation and reached a maximum at 114 h in the stationary growth phase (Fig. 4c). Non-induced cells only showed autofluorescence in the same order of magnitude as cells of the empty vector control *E. limosum* [pMTL83251]. The fluorescence intensity of lactose-induced cells was 3.5- and 3.3-fold higher compared to non-induced cells and those of the empty vector control respectively. *E.* *limosum* [pMTL83251_P_*thlA*__FAST] producing FAST constitutively showed a 1.6-fold higher fluorescence intensity comparing to that of cells with induced *feg* gene expression (Fig. 4f). Although cultivation of *E. limosum* [pMTL83251_P_*bgaL*__FAST] resulted in fluorescence, cells cultivated using glucose led to a 13.3-fold enhanced fluorescence intensity compared to cells cultivated on methanol.

Also, tdFAST2 was codon optimized for *E. limosum* and the respective gene used to construct the plasmid pMTL83251_P_*bgaL*__tdFAST2 harboring the gene *tdFAST2-opt* under the control of the *bgaR*-P_*bgal*_ lactose-inducible promoter (Fig. 4e). The induced expression of the codon-optimized *tdFAST2-opt* gene with *E. limosum* [pMTL83251_P_*bgaL*__tdFAST2] resulted in a 1.5-fold improved fluorescence compared to *E. limosum* [pMTL83251_P_*bgaL*__FAST] expressing *feg* controlled by the same promoter (Fig. 4f). The non-induced cells of *E. limosum* [pMTL83251_P_*bgaL*__tdFAST2] only showed autofluorescence.

Moreover, the fluorescence intensity of recombinant *E. limosum* strains expressing *feg* or *tdFAST2-opt* was examined when supplemented with the red fluorescent dye ^TF^Coral after excitation at 516 nm (Fig. 4f). The use of the fluorogen ^TF^Coral caused clear fluorescence of *E. limosum* [pMTL83251_P_*bgaL*__FAST] after induction of *feg* gene expression and showed 3- and 2.6-fold higher intensities compared to non-induced cells and those harboring the empty vector control, respectively. Furthermore, induced *E.* *limosum* [pMTL83251_P_*bgaL*__tdFAST2] cells showed a 1.5-fold higher fluorescent intensity compared to induced cells of *E. limosum* [pMTL83251_P_*bgaL*__FAST] in presence of ^TF^Coral. Non-induced as well as cells of the empty vector control showed autofluorescence only.

### Butanol production using FAST-tagged AdhE2 fusion proteins by recombinant *E. limosum* strains

FAST was fused to the C- or the N-terminus of AdhE2 (CA_P0035) using a flexible glycine linker. The resulting fusion proteins were used to examine the impact of the fluorescent FAST tag on functionality and productivity of AdhE2. Both AdhE2 fusion proteins were heterologously produced using the strains *E.* *limosum* [pMTL83251_P_*bgaL*__C-FAST-AdhE2] (C-terminal tag) and *E. limosum* [pMTL83251_P_*bgaL*__N-FAST-AdhE2] (N-terminal tag). *E. limosum* [pMTL83251_P_*bgaL*__AdhE2] produced the native AdhE2 from *C. acetobutylicum* and was constructed and served as a control for butanol production using glucose or methanol as a carbon source.

**Fig. 5:**
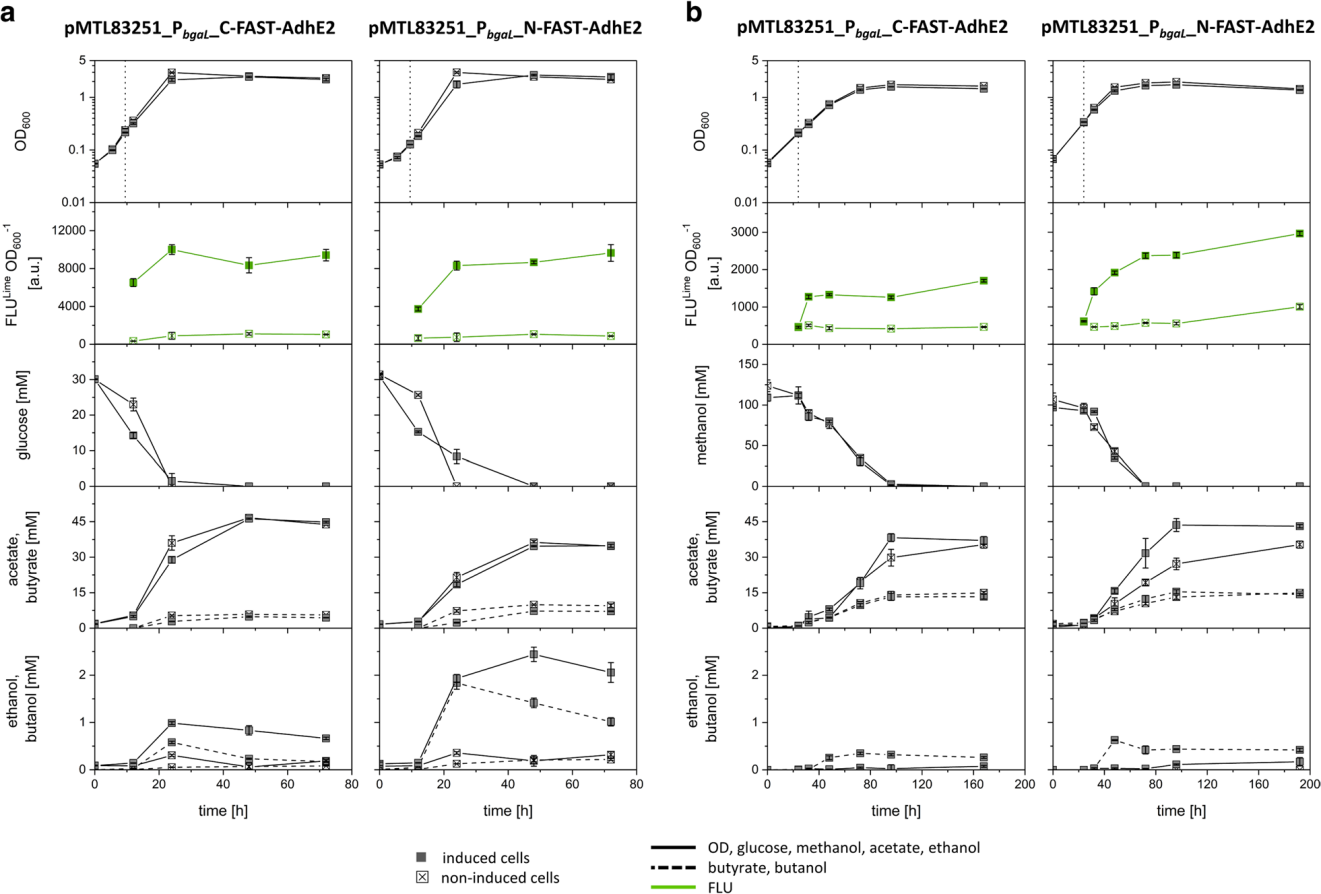
Growth experiment with *E. limosum* [pMTL83251_P_*bgaL*__C-FAST-AdhE2] and *E. limosum* [pMTL83251_P_*bgaL*__N-FAST-AdhE2]. Cells were cultivated using **a** 30 mM glucose or **b** 100 mM methanol. Gene expression of cells was either induced by lactose or non-induced. Time of induction with lactose is indicated with the vertical dotted line. OD_600_, fluorescence intensity in presence of ^TF^Lime, glucose or methanol consumption as well as acetate, butyrate, ethanol, and butanol production were monitored. Error bars indicate standard deviations. n = 3

Cultivation of *E.* *limosum* [pMTL83251_P_*bgaL*__C-FAST-AdhE2] using glucose as a carbon source and induced gene expression resulted in a maximal OD_600_ of 2.5 and a growth rate of 0.16 h^-1^. Non-induced cells reached an OD_600_ of 3 with a growth rate of 0.17 h^-1^. Clear fluorescence was observed after induction of gene expression, proving production of the C-terminal FAST-tagged AdhE2 fusion protein. Fluorescence increased during growth and reached a maximum in the late exponential growth phase. Non-induced cells showed only autofluorescence. The acetate:butyrate ratio was 10:1 and 8:1 for induced and non-induced cells, respectively. Butanol and ethanol were produced after induction of gene expression of the gene encoding the C-terminal FAST-tagged AdhE2 fusion protein. Induced cells produced up to 1 mM ethanol and 0.6 mM butanol at the end of the exponential growth phase. Non-induced cells produced traces of ethanol (0.3 mM), while no butanol was detected (Fig. 5a, Table 2, and Additional file 1: Table S1).

**Table 2:** Growth characteristics, ethanol, and butanol production of recombinant *E. limosum* strains characterized in growth experiments using glucose as carbon source

Recombinant *E. limosum* strains	OD_max_	Growth rate (h^−1^)	Ethanol (mM)	Butanol (mM)
pMTL83251_P_*bgaL*__AdhE2				
Induced	2.5	0.17	1.9	1.6
Non-induced	2.8	0.2	0.3	0.3
pMTL83251_P_*bgaL*__C-FAST-AdhE2				
Induced	2.5	0.16	1	0.6
Non-induced	3	0.17	0.3	0
pMTL83251_P_*bgaL*__N-FAST-AdhE2				
Induced	2.7	0.18	2.4	1.8
Non-induced	3	0.22	0.4	0.2

*E.* *limosum* [pMTL83251_P_*bgaL*__C-FAST-AdhE2] cultivated using methanol as a carbon source showed a growth rate of 0.05 h^-1^ with induced and non-induced gene expression and reached OD_600_ values of 1.6 and 1.8, respectively. Clear fluorescence was determined after induction of gene expression that caused production of the FAST-tagged AdhE2 fusion protein and resulted in a 3.7-fold higher intensity compared to autofluorescence of non-induced cells. The induced culture showed an acetate:butyrate ratio of 3:1, while the non-induced culture showed a ratio of 2:1. Only the induced culture of *E.* *limosum* [pMTL83251_P_*bgaL*__C-FAST-AdhE2] produced traces of ethanol (0.1 mM) and up to 0.4 mM butanol from methanol at the end of the exponential growth phase. Non-induced strains did not produce any alcohols (Fig. 5c, Table 3, and Additional file 1: Table S2).

**Table 3:** Growth characteristics, ethanol, and butanol production of recombinant *E. limosum* strains characterized in growth experiments using methanol as carbon source

Recombinant *E. limosum* strains	OD_max_	Growth rate (h^−1^)	Ethanol (mM)	Butanol (mM)
pMTL83251_P_*bgaL*__AdhE2				
Induced	1.5	0.05	0.1	0.6
Non-induced	1.7	0.05	0	0
pMTL83251_P_*bgaL*__C-FAST-AdhE2				
Induced	1.6	0.05	0.1	0.4
Non-induced	1.8	0.05	0	0
pMTL83251_P_*bgaL*__N-FAST-AdhE2				
Induced	1.7	0.06	0.2	0.6
Non-induced	2	0.06	0	0

*E.* *limosum* [pMTL83251_P_*bgaL*__N-FAST-AdhE2] cells producing the N-terminal FAST-tagged AdhE2 fusion protein, showed distinct fluorescence after induction of gene expression when cultivated with glucose, and reached a maximal OD_600_ of 2.7 with a growth rate of 0.18 h^−1^. Non-induced cells showed autofluorescence only and reached a maximal OD_600_ of 3 with a growth rate of 0.22 h^−1^. Fluorescence intensities were comparable to those determined for *E.* *limosum* [pMTL83251_P_*bgaL*__C-FAST-AdhE2] cultivated using glucose. An acetate:butyrate ratio of 5:1 was determined for induced and 4:1 for non-induced cells. Up to 2.4 mM ethanol and 1.8 mM butanol were produced at the end of the exponential growth phase with cells that expressed the gene encoding the N-terminal FAST-tagged AdhE2 fusion protein. Moreover, the uninduced cells produced up to 0.4 mM ethanol and 0.2 mM butanol (Fig. 5b, Table 2, and Additional file 1: Table S1).

*E.* *limosum* [pMTL83251_P_*bgaL*__N-FAST-AdhE2] cultivated using methanol showed a growth rate of 0.06 h^-1^, while the induced and non-induced cells reached an OD_600_ of 1.7 and 2, respectively. Induced cells produced the N-terminal FAST-tagged AdhE2 fusion protein after induction of gene expression and showed distinct fluorescence during growth with a maximum at the early stationary stage. Non-induced cells only showed autofluorescence. The acetate:butyrate ratio shifted from 3:1 to 2:1 for the induced and non-induced cells, respectively. Consequently, that strain produced 0.2 mM of ethanol and 0.6 mM of butanol at the end of the exponential growth phase. Non-induced cells again did not produce any alcohols (Fig. 5d, Table 3, and Additional file 1: Table S2). Finally, the strain that produced the N-terminal tagged version of AdhE2 showed a 1.4-fold higher fluorescence intensity compared to the strain that produced the C-terminal tagged version.

The control culture *E. limosum* [pMTL83251_P_*bgaL*__AdhE2] that produced the non-tagged version of AdhE2 showed similar characteristics regarding growth rate and production patterns. Cells were grown with glucose as a carbon source and reached a maximal OD_600_ of 2.5 with a growth rate of 0.17 h^−1^ when *adhE2* gene expression was induced. Non-induced cells showed improved growth with a maximal OD_600_ of 2.8 and a growth rate of 0.2 h^-1^. The acetate:butyrate ratio was 6:1 in the case of the induced and 5:1 in the case of the non-induced cells. Gene expression of *adhE2* in an inducible manner resulted in the production of up to 1.9 mM ethanol and 1.6 mM butanol at the end of the exponential growth phase. Non-induced cells only produced traces of ethanol and butanol (0.3 mM) (Additional file 1: Fig. S1A, Table S1, Table 2). Both, the induced as well as the non-induced cells of *E. limosum* [pMTL83251_P_*bgaL*__AdhE2] were cultivated with methanol and showed a growth rate of 0.05 h^-1^ with maximal OD_600_ values of 1.5 and 1.7, respectively. The acetate:butyrate ratio shifted to 3:1 for the induced and 2:1 for the non-induced cells. Induced expression of *adhE2* caused production of 0.6 mM butanol and traces of ethanol (0.1 mM) in the late exponential growth phase during these batch experiments. The non-induced cells produced no alcohols from methanol (Additional file 1: Fig. S1B, Table S2, Table 3).

### Acetone production with FAST-tagged Adc fusion proteins using recombinant *E. limosum* strains

The fluorescent FAST tag had no obvious negative effect on the catalytic properties of AdhE2. Subsequently, *feg* was fused to *adc* and the respective gene was expressed constitutively, together with the remaining genes of the APO, using the promoter P_*thlA*_. Thus, the two strains designated *E. limosum* [pMTL83251_P_*thlA*__C-FAST-Adc] and *E.* *limosum* [pMTL83251_P_*thlA*__N-FAST-Adc] were constructed that produced either the C- or N-terminal FAST-tagged Adc fusion protein, respectively. Those constructed strains were used to perform growth experiments to prove the functionality and productivity of the FAST-tagged Adc fusion proteins.

**Fig. 6:**
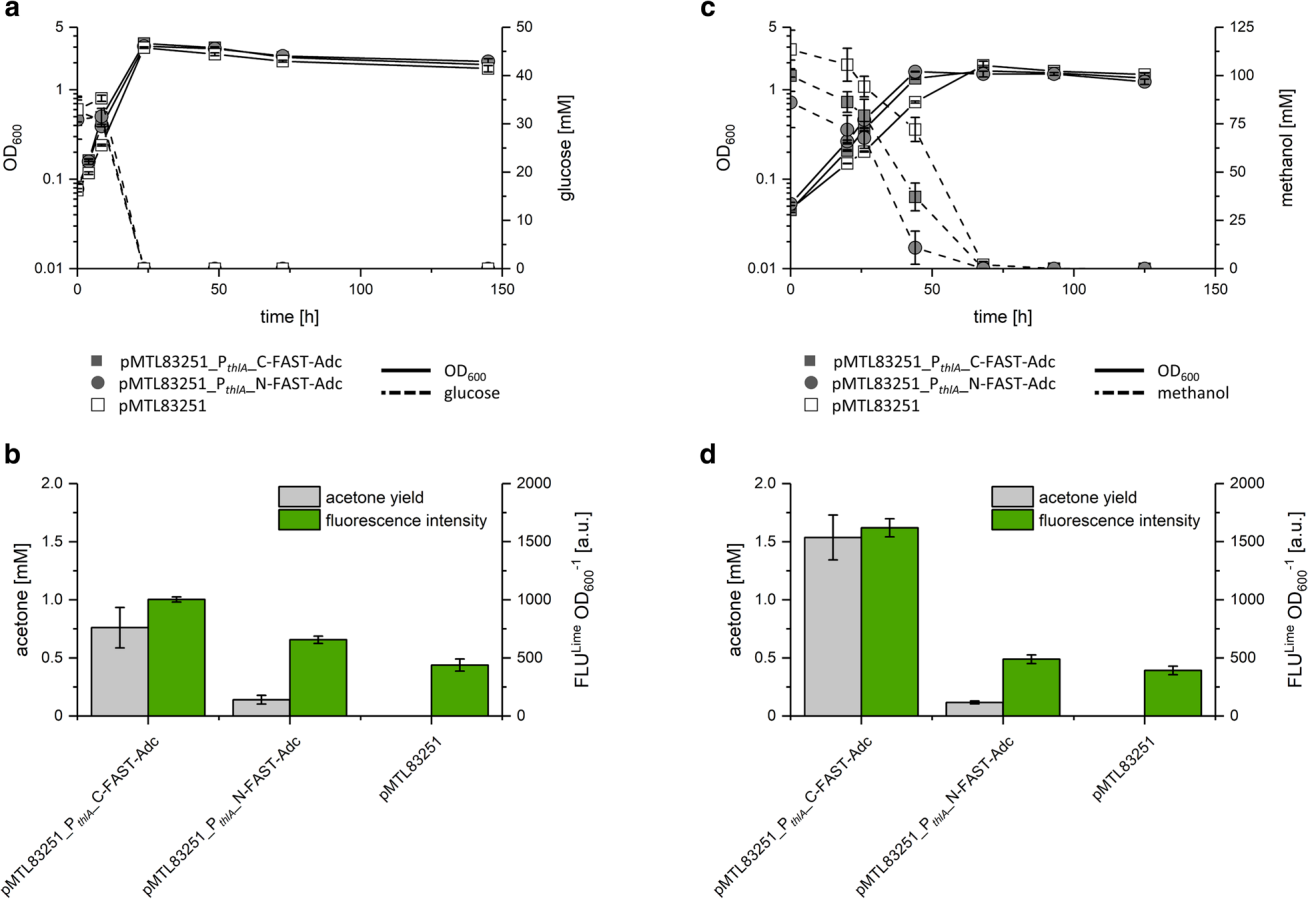
Growth experiment using *E. limosum* [pMTL83251*_*P_*thlA*_*_*C-FAST-Adc], *E. limosum* [pMTL83251_P_*thlA*__N-FAST-Adc], and *E. limosum* [pMTL83251]. Cells were cultivated using **a**, **b** 30 mM glucose or **c**, **d** 100 mM methanol as carbon source. Displayed are **a**, **c** OD_600_ and substrate consumption as well as **b**, **d** maximal acetone concentrations and fluorescence intensities. Error bars indicate standard deviations. n = 3

*E. limosum* [pMTL83251_P_*thlA*__C-FAST-Adc] and *E. limosum* [pMTL83251_P_*thlA*__N-FAST-Adc] were cultivated with glucose and showed growth rates of about 0.15 h^-1^ while reaching maximal OD_600_ values of 3.3 and 3.1, respectively (Fig. 6a and Table 4). Both strains produced acetone, acetate, and butyrate as metabolic end products (Additional file 1: Table S1 and Table 4). *E. limosum* [pMTL83251_P_*thlA*__C-FAST-Adc] cells that constitutively expressed the APO genes produced 0.8 mM of acetone, while fluorescence intensity was 2.3-fold higher compared to the autofluorescence of the empty vector control strain. *E. limosum* [pMTL83251_P_*thlA*__N-FAST-Adc] cells that expressed the gene encoding the N-terminal FAST-tagged Adc fusion protein produced only traces of acetone (0.1 mM) and exhibited 1.5-fold higher fluorescence compared to the autofluorescence of the empty vector control strain (Fig. 6b and Table 4). The control strain *E. limosum* [pMTL83251_P_*thlA*__act], expressing the non-FAST-tagged native genes of the APO controlled by the P_*thlA*_ promoter, reached an OD_600_ of 3, had a growth rate of 0.15 h^−1^, and produced acetate, butyrate as well as 0.8 mM acetone (Additional file 1: Fig. S2A and Table 4). The empty vector control *E. limosum* [pMTL83251] had a maximal growth rate of 0.17 h^-1^, reached a maximal OD_600_ of 2.5, showed only autofluorescence, and did not produce any acetone.

**Table 4:** Growth characteristics and acetone production of recombinant *E. limosum* strains characterized in growth experiments using glucose or methanol as carbon source

Recombinant *E. limosum* strains	Cultivated using glucose			Cultivated using methanol		
	OD_max_	Growth rate (h^−1^)	Acetone (mM)	OD_max_	Growth rate (h^−1^)	Acetone (mM)
pMTL83251_P_*thlA*__act	3	0.15	0.8	1.7	0.06	0.3
pMTL83251_P_*thlA*__C-FAST-Adc	3.3	0.15	0.8	1.6	0.08	1.6
pMTL83251_P_*thlA*__N-FAST-Adc	3.1	0.15	0.1	1.6	0.07	0.1

Cultivation of *E. limosum* [pMTL83251_P_*thlA*__C-FAST-Adc] and *E. limosum* [pMTL83251_P_*thlA*__N-FAST-Adc] using methanol as a carbon source resulted in maximal growth rates of 0.08 and 0.07 h^-1^, respectively (Fig. 6c and Table 4). Both cultures reached a maximal OD_600_ of 1.6 and produced acetate, butyrate, and acetone (Additional file 1: Table S2 and Table 4). *E. limosum* [pMTL83251_P_*thlA*__C-FAST-Adc] produced the highest amount of acetone (1.6 mM) cultivated using methanol, while the strain *E. limosum* [pMTL83251_P_*thlA*__N-FAST-Adc] expressing the gene encoding N-terminal tagged Adc fusion protein only produced 0.1 mM acetone. Fluorescence intensity of *E.* *limosum* [pMTL83251_P_*thlA*__C-FAST-Adc] was four- and three-fold higher compared to the fluorescence of *E. limosum* [pMTL83251] and *E. limosum* [pMTL83251_P_*thlA*__N-FAST-Adc], respectively (Fig. 6d). *E. limosum* [pMTL83251_P_*thlA*__C-FAST-Adc] cultivated using methanol showed a twofold higher acetone production as well as fluorescence intensity when compared to the cells cultivated using glucose. *E. limosum* [pMTL83251_P_*thlA*__N-FAST-Adc] showed minor fluorescence and low amounts of acetone under both conditions tested. *E. limosum* [pMTL83251_P_*thlA*__act] cultivated using methanol reached an OD_600_ of 1.7, had a growth rate of 0.6 h^-1^, and produced acetate and butyrate in a ratio of 2:1 and 0.3 mM acetone (Additional file 1: Fig S2B, Table 4). The empty vector control *E. limosum* [pMTL83251] reached a maximal OD_600_ of 1.9 with a growth rate of 0.07 h^−1^, did not produce any acetone, and only showed autofluorescence in the presence of ^TF^Lime.

## Discussion

### Methanol promotes butyrate production in *E. limosum*

Since we are aiming to produce butanol and acetone using recombinant *E. limosum* strains, different carbon sources were investigated as alternatives to commonly used sugars. Therefore, we focused on non-food feedstock-derived C1 carbon sources such as methanol, H_2_ + CO_2_, and CO containing syngas and their impact on growth behavior and product spectrum of *E.* *limosum*. Cultivation of *E. limosum* using methanol resulted in higher growth rates compared to cells cultivated with the C1 gases as reported before [43, 44]. The C1 carbon sources clearly influenced the product spectrum of *E. limosum*, since high amounts of methanol (200 mM) resulted in improved butyrate production titers. Methanol is oxidized to CO_2_ via the WLP which provides three mol NAD(P)H per methyl group oxidized [45, 47]. The NAD(P)H-NAD(P)^+^ balance is regulated during butyrate production since the reactions catalyzed by 3-hydroxybutyryl-CoA dehydrogenase and crotonase are NADH dependent and, therefore, regenerate NAD(P)^+^. Thus, the acetate:butyrate ratio shifts in favor of butyrate when cells grow on methanol [46]. Moreover, the utilization of methanol yields 1.83 ATP/butyrate, while H_2_ + CO_2_ utilization only yields 1 ATP/butyrate [47]. As shown for *C. autoethanogenum* and *A. woodii* mutants, improved ATP supply benefits fast growth and abolishes acetate production [48, 49]. In general, cultivation with methanol results in high efficiencies of bioproduction compared to the cultivation with the C1 gases [50, 51]. High amounts of methanol (200 mM) had no clear negative impact on the growth of *E. limosum*. Thus, sustainably produced methanol could be a promising substitute for glucose and C1 gases as substrate. During our experiments, the wild-type strain *E. limosum* NG-6894 did not produce any butanol. However, *E. callanderi* KIST612 (former *E. limosum* KIST612) produces low amounts of butyrate from methanol [52] as well as traces of butanol from CO [53]. Natural butanol production from C1 gases is reported for the methanol-utilizing acetogen *Butyribacterium methylotrophicum,* which is closely related to *E. limosum* [54]. In *B.* *methylotrophicum*, the production of one mol butanol from methanol requires six reducing equivalents and yields 1.5 ATP/butanol [47].

### Establishment of FAST as fluorescent reporter in *E. limosum*

We established FAST as a fluorescent reporter protein for *E. limosum* and determined the fluorescence of *feg* expressing cells using a microplate reader, fluorescence microscopy, and flow cytometry. Lactose-induced expression of *feg* caused bright fluorescence of respective *E. limosum* cells, no matter if fluorescence analytics were carried out under aerobic or anaerobic conditions. Interestingly, the *bgaR*-P_*bgaL*_ lactose-inducible system originating from *C. perfringens* was tightly regulated in *E. limosum*, since only cells with induced *feg* expression showed bright fluorescent cells. This tightly regulated expression of the *bgaR*-P_*bgaL*_ promoter was also reported for *C. perfringens* [55] *C. acetobutylicum* [56], and *C. ljungdahlii* [17], while gene expression was found to be leaky in *A. woodii* [49]. Moreover, we also showed constitutive FAST production with the P_*thlA*_ promoter from *C.* *acetobutylicum*. This constitutive expression caused improved fluorescence of cells compared to the lactose-induced *feg* expression. As of yet, only a very limited number of fluorescent proteins were shown to be somewhat functional in anaerobes, since the fluorescence of the most common fluorescent reporters including GFP and its derivatives is oxygen dependent [57]. Mostly, the flavin mononucleotide-based fluorescent proteins (FbFPs) [58] were used in anaerobes as fluorescent reporters as reported for *Clostridium cellulolyticum* [26], *Clostridioides difficile, Clostridium acetobutylicum, Clostridium sordellii* [35], *C. ljungdahlii* [59], *Clostridium beijerinckii* [60], and *Clostridium tyrobutyricum* [61]. Moreover, Shin *et al*. [13] established an engineered FbFP version (CreiLOV) as a reporter gene in *E. limosum* ATCC8486. Although FbFPs are functional under anoxic conditions, they show a fairly low brightness compared to control strains that do not express the respective fluorescent protein. In contrast, the FAST system was recently established in *C. acetobutylicum* and *C. ljungdahlii* and showed a clear and obvious fluorescence [31, 32, 62]. The fluorescence of recombinant *E. limosum* strains expressing *feg* constructed in this work is up to 26-fold improved compared to the control strain. Thus, FAST seems to be perfectly suited as a fluorescent reporter for anaerobic bacteria. An alternative to FAST could be the SNAP- and the Halo-Tag, which were shown to be functional in *C. acetobutylicum* and *C. ljungdahlii* [32]. Both tags show oxygen-independent bright fluorescence when covalently bound to a fluorogenic ligand. However, both tags require a quite long labeling time. Moreover, compared to the 14 kDa of FAST the SNAP- and Halo-Tag are larger proteins with a mass of 19.4 and 33 kDa respectively. In sum, FAST seems to be best suited for the construction of fusion proteins.

As dimerized fluorescent proteins can result in brighter fluorescence [63], we used tdFAST2 to improve fluorescence brightness with *E. limosum*. Compared to cells producing FAST, Tebo et al. [64] could show a 3.8-fold brighter fluorescence of tdFAST2-producing HEK 293 T cells. Here, we achieved a 1.5-fold improved fluorescence by expressing the codon-optimized gene encoding tdFAST2 in *E. limosum* confirming the findings of Tebo et al. [64] that production of tdFAST2 improves fluorescence brightness. With approx. 28 kDa, the molecular mass of tdFAST2 is comparable to the mass of EGFP or mCherry [65]. The improved brightness of tdFAST2 also improves the detection limit of tdFAST2-tagged proteins which might be important especially for protein localization experiments [66]. As we only showed a 1.5-fold improved fluorescence of tdFAST2, we chose the less bright but smaller FAST for the construction of AdhE2 and Adc fusion proteins.

Fluorescence microscopy of FAST-producing *E. limosum* cells revealed that plasmid-based induced expression of *feg* caused bright fluorescent *E. limosum* cells showing an inhomogeneous population. This finding was confirmed by respective flow cytometry experiments which revealed that the number of fluorescent cells varies between 72 and 59%. Such a phenotypic heterogeneity of bacterial populations with plasmid-based induced gene expression was previously reported [67–69]. One possible reason that might cause these heterogeneous populations is the usage of the lactose-inducible promoter system. The resulting *feg* gene expression may not only depend on the amount of inducer, but also on the uptake mechanism of the inducer molecule [68]. The lactose-inducible *bgaR*-P_*bgaL*_ system was also used in *C. perfringens* to express the gene *yfp-pilB* which encodes a yellow fluorescent protein-tagged fusion protein and caused an inhomogeneous fluorescent population [55]. In this experiment, Hartman et al. [55] reported that YFP-PilB was detected in 73% of the cells. Moreover, the inhomogeneous population might be caused by plasmid instability or even the loss of the plasmid encoding the reporter gene [67]. Siebert et al. [69] reported an inhomogeneous population of *Oligotropha carboxidovorans* which expressed the gene encoding mCherry in a plasmid-based manner. The reported inhomogeneity was overcome by the genomic integration of the respective gene encoding mCherry into the genome of *Oligotropha carboxidovorans*. Genomic integration of *feg* into *E. limosum* should be possible using the CRISPR-Cas9 genome editing tool [13].

Five different fluorogens are commercially available at The Twinkle Factory which can bind FAST and its variants to form the fluorescent FAST:fluorogen complex [33, 70]. Expression of *feg* or the gene encoding tdFAST2 resulted in green or red fluorescence when ^TF^Lime or ^TF^Coral was used, respectively. As different bacteria and proteins show autofluorescence upon excitation with different wavelength [34, 71, 72], FAST should be considered as a fluorescent reporter protein in a large variety of bacteria as the choice of the fluorogen can be adapted to the spectral conditions needed for the study. Recently, Tebo *et al*. [73] reported two engineered FAST versions termed greenFAST and redFAST. While greenFAST binds ^TF^Lime, redFAST only binds ^TF^Coral, which we showed can both be used in *E. limosum.* These two FAST variants augmented the FAST system and facilitate applications including two-color or even mixed culture imaging in anoxic environments.

### C- and N-terminally tagged AdhE2 and Adc fusion protein construction

In this study, we designed AdhE2 and Adc FAST-tagged fusion proteins and expressed respective genes in recombinant *E. limosum* strains. The X-ray crystal structure of Adc (3BH2) originating from *C. acetobutylicum* was provided by Ho et al. [74], but to our knowledge there is none for AdhE2 from *C. acetobutylicum*. Based on the crystal structure, first hints can be derived if either the C- or N-terminal end of the protein should be tagged without causing negative impacts regarding the folding, activity, and productivity of the enzyme. The probability of creating a functional fusion protein was maximized as both the C- and N- terminal tagged versions of both Adc and AdhE2 were constructed [75]. This approach diminishes the risk of constructing non-functional fusion proteins since the interactions of the C- and N- terminal end with other structures within the enzyme are unpredictable, especially for AdhE2. We further used a flexible GS linker as it was shown to promote proper folding of the enzyme and improves the stability of fusion proteins [76]. We cloned the native genes and genes encoding the FAST-tagged fusion proteins without affecting non-coding regions including promoters. As a result, the native genes as well as the genes encoding the FAST-tagged fusion proteins were expressed under the control of the same promoter. We consequently assume that native and tagged genes are transcribed at comparable levels.

The increasing fluorescence of *E.* *limosum* [pMTL83251_P_*bgaL*__C-FAST-AdhE2] and *E.* *limosum* [pMTL83251_P_*bgaL*__N-FAST-AdhE2] during growth proves the production of the C- and N-terminal tagged AdhE2 fusion proteins after induction of gene expression. Butanol production with strains producing the C- and N-terminal tagged AdhE2 fusion proteins prove that AdhE2 is still functional even with the fluorescent tag. The functionality of the N-terminal tagged version of AdhE2 seems not to be affected since the respective strain produced similar amounts of alcohols as the strain expressing the non-tagged native *adhE2* of *C. acetobutylicum*. However, the productivity of the C-terminal tagged version seems slightly reduced since less alcohol was produced compared to the strains just expressing *adhE2*. In addition to butanol, ethanol was produced, since AdhE2 converts not only butyryl-CoA to butanol but also acetyl-CoA to ethanol via the respective aldehyde [77]. Interestingly, if cells of both strains were cultivated with glucose as carbon source, fluorescence of the N- and C-terminal tagged fusion proteins was improved compared to cultivation on methanol. This also indicates an improved production of the fusion proteins. Moreover, both strains cultivated on glucose produced more butanol as well as ethanol compared to the cells of the respective strain cultivated using methanol. This finding indicates a coherence between the fluorescence of cells expressing genes encoding *adhE2* fusion proteins and the amount of recombinantly produced product.

In the case of the constitutive production of FAST-tagged Adc fusion proteins, only the C-terminal FAST-tagged version of Adc produced by *E.* *limosum* [pMTL83251_P_*thlA*__C-FAST-Adc] showed clear fluorescence. The strain *E.* *limosum* [pMTL83251_P_*thlA*__N-FAST-Adc] producing the N-terminal tagged version of Adc only showed a low fluorescence intensity and acetone productivity. Several factors might affect the fluorescence brightness of fluorescent fusion proteins including intrinsic brightness, folding efficiency, and translation efficiency [78]. Adc itself forms a homododecameric complex with a total mass of 365 kDa [74]. We assume, based on the X-ray structure, that the free C-terminal end of the non-tagged Adc subunits is not interfering with any residues of the protein and, therefore, not harming folding efficiency. However, the N-terminal end of Adc seems to form hydrogen bonds with LYS45, ARG44, VAL47, and GLU49 of the closest subunit. Therefore, the fusion of FAST with the sterically hindered N-terminal end might indeed affect the folding efficiency of the fusion protein or even prevents the formation of the homododecameric complex [79]. In contrast, the folding efficiency of the C-terminal FAST-tagged Adc does not seem to be affected as cells producing the respective fusion protein are fluorescent. Heterologous expression of *adc* originating from *C. acetobutylicum* and the FAST-tagged variants with *E.* *limosum* might result in inefficient translation regarding their different codon usage [80]. Adapting *adc* as well as genes encoding respective FAST-tagged Adc fusion proteins to the codon usage of *E. limosum* might improve translation efficiency causing enhanced protein production and improved fluorescence brightness. Nevertheless, *E.* *limosum* [pMTL83251_P_*thlA*__C-FAST-Adc] produced up to 0.8 mM acetone when cultivated with glucose and 1.6 mM from methanol providing evidence that the C-terminal tagged Adc fusion protein is functional and forms the catalytically active homododecameric Adc complex. *E. limosum* [pMTL83251_P_*thlA*__act] expressing the native genes of the APO produced 0.8 mM and 0.3 mM acetone when cultivated with glucose or methanol, respectively. This finding shows that the productivity of the C-terminal tagged Adc fusion protein is not negatively affected. To our knowledge, acetone was neither produced with *E.* *limosum* nor from the sustainable C1 carbon source methanol before. Although acetone production from methanol seems to be promising, several improvements of this recombinant pathway must be performed including codon harmonization, genomic integration, and bioreactor experiments which usually show a beneficial effect on bioproduction.

## Conclusions

In this study, we implemented the fluorescent activating and absorption shifting tag as an oxygen-independent fluorescent reporter protein in *E. limosum*. We showed that this reporter protein is functional under various growth conditions and can be adapted to the natural spectral properties of the organism using different fluorogens. We showed that the fluorescence of *feg*-expressing cells can be determined using a microplate reader, fluorescence microscopy, or flow cytometry. Butanol, as well as acetone, was produced by expressing the native genes of *C. acetobutylicum* and genes encoding fluorescent FAST-tagged fusion proteins. The expression of the genes encoding the fluorescent FAST-tagged fusion proteins enabled to monitor protein production during growth. We showed that those fusion proteins are functional and that the productivity is not negatively affected, however, dependent on which end the protein was tagged. The use of FAST as a fluorescence reporter not only expands the molecular toolbox of *E. limosum* but also is a promising reporter tool for other anaerobic bacteria as well. For the first time, we could show the production of the biocommodities acetone and butanol from methanol with recombinant *E. limosum* strains.

## Methods

### Strains, medium, and cultivation

The strains used in this study are listed in Table 5. *E. coli* DH5α was used for plasmid cloning and cultivated in liquid modified lysogeny broth (1% tryptone, 1% NaCl, 0.5% yeast extract) [81] while shaking or on respective agar plates at 37 °C. When required medium was supplemented with 250 µg mL^−1^ erythromycin. *E. limosum* NG-6894 was cultivated in modified DSM 135 medium under strictly anaerobic conditions at 37 °C. The modified DSM 135 medium contained 12.9 mM KH_2_PO_4_, 48.5 mM K_2_HPO_4_, 18.7 mM NH_4_Cl, 49.6 mM NaCl, 58.9 mM KHCO_3_, 2.8 mM L-cysteine-HCl, 1.5 mM MgSO_4_, 4.4 µM resazurin, 0.2% (wt/vol) yeast extract, 0.1% (vol/vol) trace element solution SL-9 [82], 0.1% (vol/vol) selenite-tungstate solution [82], and 0.2% (vol/vol) vitamin solution DSM 141. If necessary, the medium was supplemented with antibiotics (5 µg mL^-1^ clarithromycin) after autoclaving. Heterotrophic cultivations of *E. limosum* was carried out in 50 mL medium in 125 mL Müller–Krempel flasks and were supplemented with 30 mM glucose or 50, 100 or 200mM methanol. Autotrophic cultivations were also carried out in 50 mL medium in 500 mL Müller–Krempel flasks, which were pressurized to 1.1 bar overpressure with H_2_ + CO_2_ (67% H_2_ + 33% CO_2_) or syngas (10% CO_2_, 40% CO, 10% N_2_, 40% H_2_) and repressured when below 0.5 bar. Before growth experiments were carried out, cells were grown from DMSO stock cultures in 5 mL of the respective medium. These 5 mL cultures were used to prepare 50 mL precultures supplemented with glucose or methanol for subsequent growth experiments. For autotrophic growth experiments, cells from 5 mL cultures were adapted to growth conditions by transferring them two times to fresh 50 mL medium in 500 mL Müller–Krempel culture flasks with the respective gas atmosphere.

**Table 5:** Strains used in this study

Strain	Description	Source
*E. coli* DH5α	*E. coli* for plasmid construction	Thermo Fisher Scientific Inc., Waltham, MA, USA
*E. limosum* NG-6894	This strain grows on a defined medium (without any yeast extract) and does not produce any sticky polymers	Prof. Philippe Soucaille, INSA, University of Toulouse, France

### Plasmid construction

All plasmids and primers used in this study are listed in Tables 6 and 7, respectively. DNA fragments for cloning purposes were amplified using the KAPA Hifi polymerase (Kapa Biosystem, Sigma-Aldrich Chemie GmbH, Munich, Germany). Primers used in this study were synthesized by biomers.net GmbH (Ulm, Germany) and designed to have a 15–25 nucleotide overlap with the respective vector DNA. Vector DNA for cloning purposes was linearized using Fast digest enzymes (Thermo Fisher Scientific Inc., Waltham, USA). PCR and digested vector DNA fragments were purified using the NucleoSpin**®** Gel and PCR clean-up kit (Macherey-Nagel GmbH & Co. KG, Düren, Germany). All plasmids were constructed by assembling PCR and vector DNA fragments using the NEBuilder Hifi DNA Assembly Cloning Kit according to the manufacturer’s protocol (New England Biolabs, Ipswich, MA, USA). Assembled plasmids were used for the transformation of chemically competent *E. coli* DH5α cells and verified via Sanger sequencing (Eurofins Genomics GmbH, Luxemburg).

To establish FAST as a reporter protein in *E. limosum*, plasmids harboring *feg* were constructed. Therefore, *feg* was amplified together with the promoter P_*thl*_^*sup*^ from plasmid p95thl^sup^FAST [31] using primers FW_Pthlsup_FAST_NdeI and RV_Pthlsup_FAST_XhoI (Table 7). The amplified PCR fragment was cloned via *Nde*I and *Xho*I recognition sites into vector pMTL83251 resulting in the plasmid pMTL83251_P_*thl*_^*sup*^_FAST. The promoter region of P_*thl*_^*sup*^ from plasmid pMTL83251_P_*thl*_^*sup*^_FAST was further exchanged with the lactose-inducible *bgaR*-P_*bgaL*_ system from *C. perfringens* [55] and P_*thlA*_ from *C. acetobutylicum* [18]. Therefore, the *bgaR*-P*bgaL-*encoding DNA fragment was amplified from plasmid pMTL83151_gusA_P_*bgaL*_ [49] using the primers FW_PbgaL_NdeI and RV_PbgaL_BamHI. The DNA fragment containing the promoter region of P_*thlA*_ was amplified from plasmid pJIR750_act [18] using primers FW_Pthl_NdeI and RV_Pthl_BamHI (Table 7). Both promoter containing DNA fragments were assembled with pMTL83251_P_*thl*_^*sup*^_FAST (excision of P_*thl*_^*sup*^) resulting in plasmids pMTL83251_P_*thlA*__FAST and pMTL83251_P_*bgaL*__FAST.

The gene *feg* of pMTL83251_P_*bgaL*__FAST was exchanged with the gene encoding tdFAST2. The sequence of tdFAST2 was obtained from Tebo et al. [64] and codon optimized for *E. limosum* using the GENEius tool (Eurofins Genomics GmbH, Luxemburg) and synthesized by Eurofins Genomics Germany GmbH (Ebersberg, Germany). The gene *tdFAST2-opt* was amplified from the delivered plasmid pEX-A128_tdFAST2-opt using the primers FW_tdFAST2-opt_BamHI and RV_tdFAST2-opt_XhoI (Table 7) and ligated with *Bam*HI and *Xho*I digested pMTL83251_P_*bgaL*__FAST (excision of *feg*) vector DNA resulting in plasmid pMTL83251_P_*bgaL*__tdFAST2.

For butanol production with *E. limosum,* the plasmid pMTL83251_P_*bgaL*__AdhE2 was constructed. The gene *adhE2* was amplified from genomic DNA of *C. acetobutylicum* using primers FW_C1_adhe2 and RV_N2_adhE2 (Table 7). The gene was assembled under the control of the lactose-inducible *bgaR*-P_*bgaL*_ promoter using the plasmid DNA of pMTL83251_P_*bgaL*__FAST, which was digested using *Bam*HI and *Xho*I (excision of *feg*).

For acetone production with *E. limosum,* the plasmid pMTL83251_P_*thlA*__act was constructed encoding the genes of the APO under the control of promoter P_*thlA*_. The origin of these genes as well as their locus tags are listed in Table 6. These genes as well as the DNA fragment containing the promoter region of P_*thlA*_ were amplified from plasmids pJIR750_act using primers FW_act_NdeI and RV_act_XhoI (Table 7). The amplified PCR fragments were cloned via the *Nde*I and *Xho*I restriction sites of pMTL83251 resulting in the plasmids pMTL83251_P_*thlA*__act.

**Table 6:** Plasmids used in this study

Plasmid	Description	Source
pMTL83251	ColE1 ori^-^, pCB102 ori^+^, Em^r^, *traJ*, *lacZ*	[83]
pMTL83151_gusA_P_*bgaL*_	ColE1 ori^-^, pCB102 ori^+^, Cm^r^, *traJ*, *lacZ*, *gusA* from *E. coli*, *bgaR-*P_*bgaL*_ from *C. perfringens*	[49]
pJIR750_act	pJIR750; P_*thl*_, *thlA* (CAC2873), *ctfA/B* (CA_P0163/P0164), *adc* (CA_P0165) from *C. acetobutylicum*	[18]
p95thl^sup^FAST	ColE1 ori^-^, *repL* ori^+^, Amp^r^, Em^r^, *lacZ,* P_*thl*_^*sup*^*, feg*	[31]
pMTL83251_P_*thl*_^*sup*^_FAST	pMTL83251; P_*thl*_^*sup*^, *feg* from p95thl^sup^FAST	This work
pMTL83251_P_*thlA*__FAST	pMTL83251; P_*thlA*_, from pMTL83251_P_*thlA*__act; *feg* from p95thl^sup^FAST	This work
pMTL83251_P_*bgaL*__FAST	pMTL83251; *bgaR-*P_*bgaL*_ from *C. perfringens*; *feg* from p95thl^sup^FAST	This work
pEX-A128_tdFAST2-opt	pUC ori^-^, Amp^r^, MSC, gene encoding tdFAST2 codon optimized for *E. limosum*	Eurofins Genomics Germany GmbH, Ebersberg, Germany
pMTL83251_P_*bgaL*__tdFAST2	pMTL83251; *bgaR-*P_*bgaL*_ from *C. perfringens*; gene encoding tdFAST2 codon optimized for *E. limosum*	This work
pMTL83251_P_*bgaL*__AdhE2	pMTL83251; *bgaR-*P_*bgaL*_ from *C. perfringens*; *adhE2* from *C. acetobutylicum*	This work
pMTL83251_P_*bgaL*__C-FAST-AdhE2	pMTL83251; *bgaR-*P_*bgaL*_ from *C. perfringens*; *adhE2* from *C. acetobutylicum* C-terminal tagged with *feg* from p95thl^sup^FAST	This work
pMTL83251_P_*bgaL*__N-FAST-AdhE2	pMTL83251; *bgaR-*P_*bgaL*_ from *C. perfringens*; *adhE2* from *C. acetobutylicum* N-terminal tagged with *feg* from p95thl^sup^FAST	This work
pMTL83251_P_*thlA*__act	pMTL83251; P_*thl*_, *thlA*, *ctfA/B*, *adc* from pJIR_act	This work
pMTL83251_P_*thlA*__C-FAST-Adc	pMTL83251; P_*thlA*_, *thlA*, *ctfA/B*, from pJIR_act; *adc* from *C. acetobutylicum* C-terminal tagged with *feg* from p95thl^sup^FAST	This work
pMTL83251_P_*thlA*__N-FAST-Adc	pMTL83251; P_*thlA*_, *thlA*, *ctfA/B*, from pJIR_act; *adc* from *C. acetobutylicum* N-terminal tagged with *feg* from p95thl^sup^FAST	This work

**Table 7:** Primers used in this study

Primer	Sequence 5′–3′	Length (bp)
FW_C1_adhE2	ttaaatgtattgggagggtggatccatgaaagttacaaatcaaaaagaac	50
RV_N2_adhE2	agcttgcatgtctgcaggcctcgagttaaaatgattttatatagatatccttaagttc	58
FW_act_NdeI	gaccgcggccgctgtatccatatgtcaagaagaggcac	38
RV_act_XhoI	agcttgcatgtctgcaggcctcgagttacttaagataatcatatataacttcag	54
FW_Pthlsup_FAST_NdeI	atgaccgcggccgctgtatccatatgtttttaacaaaaagtattgaaatttg	52
RV_Pthlsup_FAST_XhoI	aagcttgcatgtctgcaggcctcgagtcataccctcttaac	41
FW_PbgaL_NdeI	gaccgcggccgctgtatccatatgtaatttagatattaattctaaattaagtgaaattaatatag	65
RV_PbgaL_BamHI	caaatgctacgtgttccatggatccaccctcccaatacatttaaaataattatg	54
FW_Pthl_NdeI	gaccgcggccgctgtatccatatgtcaagaagaggcacctcatc	44
RV_Pthl_BamHI	caaatgctacgtgttccatggatcctaacctcctaaattttgatacgg	48
FW_tdFAST2-opt_BamHI	ttaaatgtattgggagggtggatccatggagcatgtagcttttg	44
RV_tdFAST2-opt_XhoI	agcttgcatgtctgcaggcctcgagtcaaacccgtttgacgaag	44
RV_C1_adhE2-FAST	ctacgtgttcagaaccaccaccaccaaatgattttatatagatatccttaagttc	55
FW_C2_adhE2-FAST	aaaatcatttggtggtggtggttctgaacacgtagcatttggaag	45
RV_C2_FAST	agcttgcatgtctgcaggcctcgagtcataccctcttaacgaaaac	46
FW_N1_FAST	ttaaatgtattgggagggtggatccatggaacacgtagcatttg	44
RV_N1_FAST-adhE2	ttgtaactttagaaccaccaccacctaccctcttaacgaaaac	43
FW_N2_FAST-adhE2	taagagggtaggtggtggtggttctaaagttacaaatcaaaaagaactaaaac	53
FW_C1_adc	acccatggctgtttaggtaccttttatgttaaaggatgaagtaattaaac	50
RV_C1_adc-FAST	ctacgtgttcagaaccaccaccacccttaagataatcatatataacttcagc	52
FW_C2_adc-FAST	ttatcttaagggtggtggtggttctgaacacgtagcatttggaag	45
FW_N1_FAST-adc	acccatggctgtttaggtaccttttatggaacacgtagcatttg	44
RV_N1_FAST-adc	catcctttaaagaaccaccaccacctaccctcttaacgaaaac	43
FW_N2_FAST-adc	taagagggtaggtggtggtggttctttaaaggatgaagtaattaaacaaattag	54
RV_N2_adc	agcttgcatgtctgcaggcctcgagttacttaagataatcatatataacttcag	54

Moreover, plasmids were constructed to produce C- and N- terminal FAST-tagged fusion proteins of AdhE2 and Adc. FAST was C- and N-terminal fused to AdhE2 or Adc using a glycine linker (GGGGS) with the sequence ggtggtggtggttct. For the construction of C-terminal tagged fusion proteins, the 3’ end of the gene of interest was fused to the 5’ end of *feg.* Therefore, the gene of interest was amplified without its stop codon while *feg* was amplified without its start codon. To construct N-terminal FAST-tagged fusion proteins, the 3′ end of *feg* was fused to the 5′ end of the gene of interest*.* The gene of interest was amplified without its start codon, while *feg* was amplified without its stop codon.

The plasmid pMTL83251_P_*bgaL*__C-FAST-AdhE2 was constructed to produce the C-terminal FAST-tagged AdhE2 fusion protein. *adhE2* was amplified from plasmid pMTL83251_P_*bgaL*__AdhE2 using primers FW_C1_adhE2 and RV_C1_adhE2-FAST (Table 7), *feg* was amplified from plasmid pMTL83251_P_*bgaL*__FAST using primers FW_C2_adhE2-FAST and RV_C2_FAST (Table 7). Primers RV_C1_adhE2-FAST and FW_C2_adhE2-FAST contain the sequence for the glycine-linker. The plasmid pMTL83251_P_*bgaL*__N-FAST-AdhE2 was constructed to produce the N-terminal FAST-tagged AdhE2 fusion protein. *adhE2* was amplified from plasmid pMTL83251_P_*bgaL*__AdhE2 using primers FW_N1_adhE2 and RV_N1_FAST-adhE2 while *feg* was amplified from plasmid pMTL83251_P_*bgaL*__FAST using primers FW_N2_FAST-adhE2 and RV_N2_adhE2 (Table 7). Primers RV_N1_FAST-adhE2 and FW_N2_FAST-adhE2 contain the sequence for the glycine linker.

The plasmid pMTL83251_P_*thlA*__C-FAST-Adc was constructed to produce the C-terminal FAST-tagged Adc fusion protein. *adc* was amplified from plasmid pMTL83251_P_*thlA*__act using primers FW_C1_adc and RV_C1_adc-FAST, and *feg* was amplified from plasmid pMTL83251_P_*bgaL*__FAST using primers FW_C2_adc-FAST and RV_C2_FAST (Table 7). Primers RV_C1_adc-FAST and FW_C2_adc-FAST contain the sequence for the glycine linker. The plasmid pMTL83251_P_*thlA*__N-FAST-Adc was constructed to produce the N-terminal FAST-tagged Adc fusion protein. The gene *adc* was amplified from plasmid pMTL83251_P_*thlA*__act using primers FW_N1_adc-FAST and RV_N1_FAST-adc and *feg* was amplified from plasmid pMTL83251_P_*bgaL*__FAST using primers FW_N2_FAST-adc and RV_N2_adc (Table 7). Primers RV_N1_FAST-adc and FW_N2_FAST-adc contain the sequence for the glycine linker.

### Transformation

Electroporation and preparation of electrocompetent *E. limosum* cells were performed according to the protocol of Leang *et al*. [84] with several modifications. All steps were carried out under anaerobic conditions in an anaerobic chamber (gas atmosphere 95% N_2_ and 5% H_2_). Plastic materials were placed 24 h before use into the anaerobic chamber to remove any traces of oxygen. For the preparation of electrocompetent cells, *E. limosum* was cultivated at 37 °C in 100 mL modified DSM 135 medium supplemented with 100 mM methanol and 40 mM DL-Threonine. Cells were cultivated until the early exponential growth phase (OD_600_ 0.3–0.5) and harvested by centrifugation at 7690×*g* for 10 min at 4 °C. Subsequently, cells were washed two times with anaerobic SMP buffer (270 mM sucrose, 1 mM MgCl_2_, 7 mM NaH_2_PO_4_, pH 6) by centrifugation at 7.690×*g* for 10 min at 4 °C. Afterwards, the cell pellet was suspended in 648 µL SMP buffer and 72 µL DMSO, distributed into cryotubes, and stored at − 80 °C until use.

Transformation of *E. limosum* cells was performed using 3–5 µg plasmid DNA, which were added to 25 µL of electrocompetent cells into a pre-cooled 1 mm electroporation cuvette (Biozym Scientific GmbH, Oldendorf, Germany). Cells were pulsed (625 V, 25 μF, 600 Ω; Gene Pulser Xcell^TM^, Bio-Rad Laboratories GmbH, Munich, Germany) and immediately transferred into 5 mL fresh medium. *E. limosum* cells were recovered at 37 °C in 5 mL modified DSM 135 medium supplemented with 100 mM methanol. The OD_600_ of transformed cells was monitored. After one or two doublings, 5 µg mL^−1^ clarithromycin was added to the medium for selection of recombinant strains. After a further increase of the OD_600_, which indicates a successful transformation, cells were transferred two more times into fresh medium supplemented with 5 µg mL^−1^ of clarithromycin. Successfully transformed cells were verified by isolation of plasmid DNA using the Zyppy^TM^ Plasmid Miniprep Kit (Zymo Research, Irvine, CA, USA). This preparation was used to transform chemically competent *E. coli* DH5α cells to amplify the plasmids. After transformation, plasmids were isolated and checked by restriction analysis. Recombinant strains were stored in cryotubes with 10% DMSO at − 80 °C. All recombinant strains constructed in this study are listed in Table 8.

**Table 8:** Recombinant strains used in this study

Recombinant strain	Plasmid	Reference
*E. limosum* [pMTL83251]	pMTL83251	This work
*E. limosum* [pMTL83251_P_*thlA*__FAST]	pMTL83251_P_*thlA*__FAST	This work
*E. limosum* [pMTL83251_P_*bgaL*__FAST]	pMTL83251_P_*bgaL*__FAST	This work
*E. limosum* [pMTL83251_P_*bgaL*__tdFAST2]	pMTL83251_P_*bgaL*__tdFAST2	This work
*E. limosum* [pMTL83251_P_*bgaL*__AdhE2]	pMTL83251_P_*bgaL*__AdhE2	This work
*E. limosum* [pMTL83251_P_*bgaL*__C-FAST-AdhE2]	pMTL83251_P_*bgaL*__C-FAST-AdhE2	This work
*E. limosum* [pMTL83251_P_*bgaL*__N-FAST-AdhE2]	pMTL83251_P_*bgaL*__N-FAST-AdhE2	This work
*E. limosum* [pMTL83251_P_*thlA*__act]	pMTL83251_P_*thlA*__act	This work
*E. limosum* [pMTL83251_P_*thlA*__C-FAST-Adc]	pMTL83251_P_*thlA*__C-FAST-Adc	This work
*E. limosum* [pMTL83251_P_*thlA*__N-FAST-Adc]	pMTL83251_P_*thlA*__N-FAST-Adc	This work

### Fluorescence determination

Fluorescence of FAST and FAST-tagged fusion protein-producing recombinant *E.* *limosum* strains, cultivated anaerobically until the stationary growth phase, were determined using a microplate reader, fluorescence microscopy, or flow cytometry. Therefore, 2 mL culture broth was taken anaerobically during growth and harvested by centrifugation at 7711×*g* for 15 minutes at 4°C. The supernatant was discarded and harvested cells were washed with anaerobic PBS buffer (137 mM NaCl, 2.7 mM KCl, 10 mM Na_2_HPO_4_, 1.8 mM KH_2_PO_4_) followed by centrifugation at 7711×*g* for 15 minutes at 4°C. Cell pellets were suspended in anaerobic PBS buffer (end OD_600_ 1).

### Microplate reader

100 µL of suspended cells was transferred to black flat-bottomed 96-well microtiter plates (Greiner Bio-One GmbH, Frickenhausen, Deutschland) and supplemented with 10 µM fluorogen of the green dye ^TF^Lime (ex. 480/em. 541) or the red dye ^TF^Coral (ex. 516/em. 600) (The Twinkle Factory, France, Paris). Excitation and emission wavelengths both correspond to the respective maximum of the fluorogen. Fluorescence intensities of the whole population were determined using the SYNERGY H1 microplate reader (BioTek, Bad Friedrichshall, Germany) located in an anaerobic chamber and finally normalized to the OD_600_ of PBS-washed cells.

### Fluorescence microscopy

Washed cells were stained with 10 µM ^TF^Lime and transferred onto an 1% agarose pad on a microscopy slide, covered with a glass coverslip, and sealed with nail polish. Cells were viewed with a 63x objective, using a Zeiss Axio Observer Z1 (Zeiss, Oberkochen, Germany). Green fluorescence of the FAST:fluorogen complex was detected using the Zeiss filter set 38 HE (ex. BP 470/40, em. BP 525/50). Data were analyzed using the Zeiss Zen 2.6 blue edition software.

### Flow cytometry

Washed cells were stained with 10 µM ^TF^Lime diluted in PBS buffer (end OD_600_: 0.01). Green fluorescence of the FAST:fluorogen complex was assessed using an excitation wavelength of 488 nm and a 528/46 nm emission filter. For analysis, at least 10,000 events were recorded using an Amnis® CellStream® flow cytometer (Luminex Corporation, Austin, TX, USA). Acquired data were analyzed using the CellStreamTM Analysis tool version 1.2.152 (Luminex Corporation, Austin, TX, USA).

### Analytics

2 mL samples were withdrawn from cell cultures during the growth of *E. limosum* to analyze OD_600_, product spectrum as well as substrate consumption. After OD_600_ of the withdrawn culture broth was determined at 600 nm using the GENESYS 10vis photometer (Thermo Scientific, Waltham, MA, USA), the remaining culture was centrifuged at 17,968×*g* for 20 minutes at 4 °C to remove cell debris. The supernatant was used for HPLC and GC analysis.

### High-performance liquid chromatography

The concentration of glucose, acetate, and butyrate from culture supernatant was determined using the Agilent 1260 Infinity II HPLC system (Agilent Technologies, Santa Clara, CA, USA), equipped with a diode array detector and a refractive index detector. To achieve separation, 20 μL of the supernatant was injected into the organic acid resin 150 x 8 mm column (CS-chromatographie-Service GmbH, Langerwehe, Germany) packed with polystyrene divinylbenzene copolymer operating at a constant temperature of 40 °C. As mobile phase, 5 mM H_2_SO_4_ was used with a flow rate of 0.7 mL min^-1^. The software OpenLAB CDS ChemStation Edition A.01.03 (Agilent Technologies, Santa Clara, CA, USA) was used for data analysis.

### Gas chromatography

The concentration of methanol, ethanol, butanol, and acetone in the culture broth was analyzed via gas chromatography. A PerkinElmer Clarus 680 GC system (Perkin Elmer LAS GmbH, Waltham, MA, USA) equipped with an Elite-FFAP capillary column (length 30 m x inner diameter 0.32 mm, film thickness 0.25 µm) (Perkin Elmer LAS GmbH, Waltham, MA, USA) and FID detector was used. Supernatants were acidified with 2 M HCL. H_2_ was used as the carrier gas. The injector and detector were operated at 225 and 300 °C, respectively. 0.5 µL of the culture broth was injected into the gas chromatograph and analyzed using the following temperature profile: 40 °C for 2.5 minutes; 40 °C–250 °C with 40 °C min^-1^; 250 °C for 2 minutes.

## Supplementary Information

**Additional file 1.** **Fig. S1.** Growth experiment with *E. limosum* [pMTL83251_P_*bgaL*__AdhE2] and *E. limosum* [pMTL83251]. Strains were cultivated using 30 mM glucose (A) or 100 mM methanol (B) as carbon source. Gene expression of cells was either induced by lactose or non-induced. Induction with lactose is indicated with the vertical dotted line. Monitored are OD_600_, methanol consumption, as well as acetate, butyrate, ethanol, and butanol production. Error bars indicate standard deviations. n = 3. **Fig. S2.** Growth experiment with *E. limosum* [pMTL83251_P_*thlA*__act] and *E. limosum* [pMTL83251]. Strains were cultivated using 30 mM glucose (A) or 100 mM methanol (B) as carbon source. Monitored are OD_600_, glucose and methanol consumption, as well as acetate, butyrate, and acetone production. Error bars indicate standard deviations. n = 3. **Table S1.** Growth characteristics and product formation of recombinant *E. limosum* strains characterized in growth experiments using glucose as carbon source. **Table S2.** Growth characteristics and product formation of recombinant *E. limosum* strains characterized in growth experiments using methanol as carbon source.

## Data Availability

All data generated during this study are included in this article and the additional files. Raw data are available on reasonable request.

## Abbreviations

Adc: Acetoacetate decarboxylase
AdhE2: Bifunctional acetaldehyde/alcohol dehydrogenase
APO: Acetone production operon
FAST: Fluorescence-activating and absorption shifting tag
OD_600_: Optical density
POI: Protein of interest
