# Supplementary material for: Production of the biocommodities butanol and acetone from methanol with fluorescent FAST-tagged proteins using metabolically engineered strains of Eubacterium limosum
Source: Biotechnol Biofuels. 2021 May 10;14:117. doi: 10.1186/s13068-021-01966-2 (PMC8111989; doi:10.1186/s13068-021-01966-2)
Supplement: Supplementary file 1 — Additional file 1. Fig. S1. Growth experiment with E. limosum [pMTL83251_PbgaL_AdhE2] and E. limosum [pMTL83251]. Strains were cultivated using 30 mM glucose (A) or 100 mM methanol (B) as carbon source. Gene expression of cells was either induced by lactose or non-induced. Induction with lactose is indicated with the vertical dotted line. Monitored are OD600, methanol consumption, as well as acetate, butyrate, ethanol, and butanol production. Error bars indicate standard deviations. n = 3. Fig. S2. Growth experiment with E. limosum [pMTL83251_PthlA_act] and E. limosum [pMTL83251]. Strains were cultivated using 30 mM glucose (A) or 100 mM methanol (B) as carbon source. Monitored are OD600, glucose and methanol consumption, as well as acetate, butyrate, and acetone production. Error bars indicate standard deviations. n = 3. Table S1. Growth characteristics and product formation of recombinant E. limosum strains characterized in growth experiments using glucose as carbon source. Table S2. Growth characteristics and product formation of recombinant E. limosum strains characterized in growth experiments using methanol as carbon source. [file 13068_2021_1966_MOESM1_ESM.docx]

**
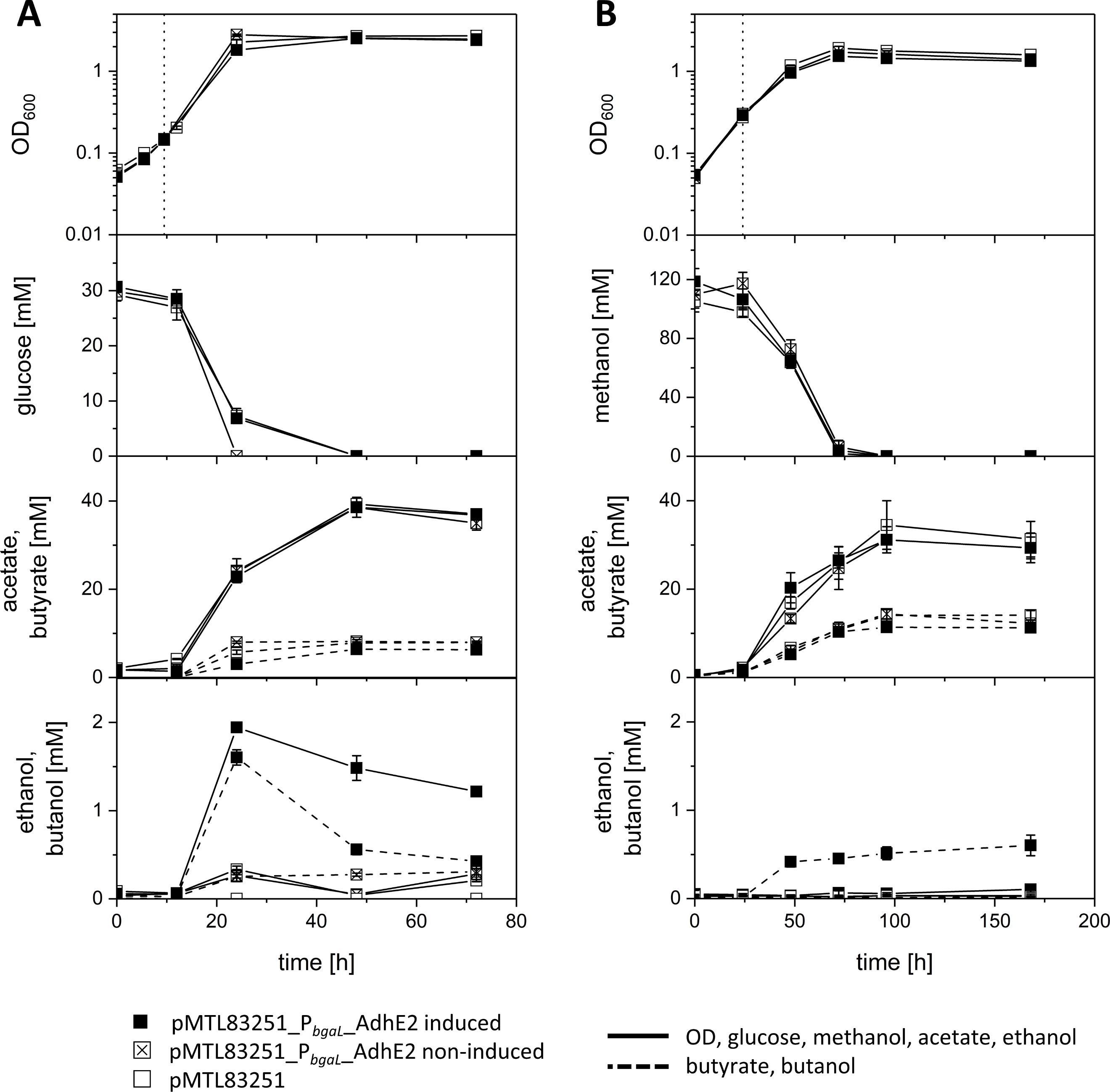
**

**Fig. S1** Growth experiment with E. limosum [pMTL83251_P_bgaL__AdhE2] and E. limosum [pMTL83251]. Strains were cultivated using (**A**) 30 mM glucose or (**B**) 100 mM methanol as carbon source. Gene expression of cells was either induced by lactose or non-induced. Induction with lactose is indicated with the vertical dotted line. Monitored are OD_600_, methanol consumption, as well as acetate, butyrate, ethanol, and butanol production. Error bars indicate standard deviations. n=3.


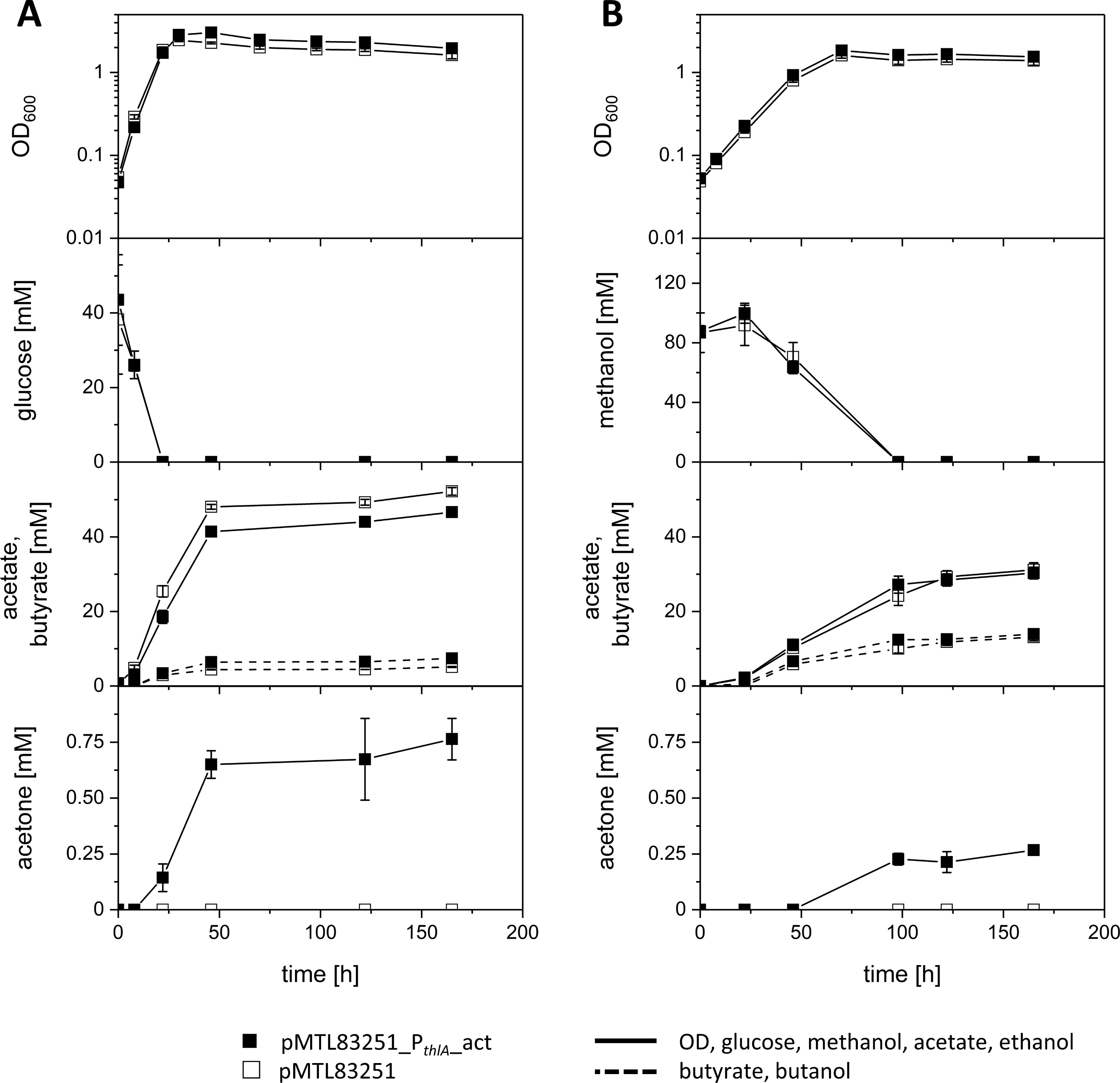


**Fig. S2** Growth experiment with E. limosum [pMTL83251_P_thlA__act] and E. limosum [pMTL83251]. Strains were cultivated using (**A**) 30 mM glucose or (**B**) 100 mM methanol as carbon source. Monitored are OD_600_, glucose and methanol consumption, as well as acetate, butyrate, and acetone production. Error bars indicate standard deviations. n=3.

**Table S1** Growth characteristics and product formation of recombinant E. limosum strains characterized in growth experiments using glucose as carbon source.

| strain | OD_max_ | growth rate µ  [h^-1^] | products [mM] | | | | | products  [mol / 100 mol substrate] | | | | | product ratio [acetate:butyrate] |
| --- | --- | --- | --- | --- | --- | --- | --- | --- | --- | --- | --- | --- | --- |
|  |  |  | acetate | butyrate | ethanol | butanol | acetone | acetate | butyrate | ethanol | butanol | acetone |  |
| pMTL83251^1^ | 2.7 | 0.19 | 39.3 | 7.9 | 0.3 | 0 | n.d. | 134.5 | 26.8 | 0 | 0 | n.d. | 5:1 |
| pMTL83251_P*_bgaL_*_AdhE2 induced | 2.5 | 0.17 | 38.6 | 6.4 | 1.9 | 1.6 | n.d. | 125.7 | 20.8 | 6.2 | 5.2 | n.d. | 6:1 |
| pMTL83251_P*_bgaL_*_AdhE2 non-induced | 2.8 | 0.2 | 38.5 | 8.2 | 0.3 | 0.3 | n.d. | 129.3 | 27.5 | 1 | 1 | n.d. | 5:1 |
| pMTL83251^2^ | 2.5 | 0.13 | 52.2 | 5.1 | n.d. | n.d. | 0 | 136.6 | 13.3 | n.d. | n.d. | 0 | 10:1 |
| pMTL83251_P*_thlA_*_act | 3.0 | 0.15 | 46.7 | 7.4 | n.d. | n.d. | 0.8 | 107.2 | 17.0 | n.d. | n.d. | 1.8 | 6:1 |
| pMTL83251_P*_bgaL_*_C-FAST-AdhE2 induced | 2.5 | 0.16 | 46.3 | 4.8 | 1 | 0.6 | n.d. | 153.7 | 16.1 | 3.3 | 1.9 | n.d. | 10:1 |
| pMTL83251_P*_bgaL_*_C-FAST-AdhE2 non-induced | 3 | 0.17 | 46.7 | 5.8 | 0.3 | 0 | n.d. | 155.3 | 19.3 | 1 | 0 | n.d. | 8:1 |
| pMTL83251_P*_bgaL_*_N-FAST-AdhE2 induced | 2.7 | 0.18 | 34.7 | 7.2 | 2.4 | 1.8 | n.d. | 112.3 | 23.4 | 7.9 | 6 | n.d. | 5:1 |
| pMTL83251_P*_bgaL_*_N-FAST-AdhE2 non-induced | 3 | 0.22 | 36.3 | 9.9 | 0.4 | 0.2 | n.d. | 115.3 | 31.5 | 1.3 | 0.7 | n.d. | 4:1 |
| pMTL83251^3^ | 2.5 | 0.17 | 35.7 | 10.9 | 0 | 0 | 0 | 101.1 | 30.9 | 0 | 0 | 0 | 3:1 |
| pMTL83251_P*_thlA_*_C-FAST-Adc | 3.3 | 0.15 | 35.9 | 9.5 | 0 | 0 | 0.8 | 109.9 | 29 | 0 | 0 | 2.3 | 4:1 |
| pMTL83251_P*_thlA_*_N-FAST-Adc | 3.1 | 0.15 | 39.7 | 9.3 | 0 | 0 | 0.1 | 125.8 | 29.4 | 0 | 0 | 0.4 | 4:1 |

^1^ control strain during growth experiment with *E. limosum* [pMTL83251_P*_bgaL_*_AdhE2]

^2^ control strain during growth experiment with *E. limosum* [pMTL83251_P*_thlA_*_act]

^3^ control strain during growth experiment with *E. limosum* [pMTL83251_P*_thlA_*_C-FAST-Adc] and *E. limosum* [pMTL83251_P*_thlA_*_N-FAST-Adc]

n.d.: not determined

**Table S2** Growth characteristics and product formation of recombinant E. limosum strains characterized in growth experiments using methanol as carbon source.

| strain | OD_max_ | growth rate µ  [h^-1^] | products [mM] | | | | | products  [mol / 100 mol substrate] | | | | | product ratio [acetate:butyrate] |
| --- | --- | --- | --- | --- | --- | --- | --- | --- | --- | --- | --- | --- | --- |
|  |  |  | acetate | butyrate | ethanol | butanol | acetone | acetate | butyrate | ethanol | butanol | acetone |  |
| pMTL83251^1^ | 1.8 | 0.06 | 34.5 | 14.1 | 0 | 0 | n.d. | 32.8 | 13.3 | 0 | 0 | n.d. | 5:2 |
| pMTL83251_P*_bgaL_*_AdhE2 induced | 1.5 | 0.05 | 31.2 | 11.4 | 0.1 | 0.6 | n.d. | 26.3 | 9.6 | 0.1 | 0.5 | n.d. | 3:1 |
| pMTL83251_P*_bgaL_*_AdhE2 non-induced | 1.7 | 0.05 | 31.2 | 14.4 | 0 | 0 | n.d. | 28.4 | 13.1 | 0 | 0 | n.d. | 2:1 |
| pMTL83251^2^ | 1.6 | 0.06 | 31.2 | 13.1 | n.d. | n.d. | 0 | 34 | 14.3 | n.d. | n.d. | 0 | 2:1 |
| pMTL83251_P*_thlA_*_act | 1.7 | 0.06 | 30.3 | 14 | n.d. | n.d. | 0.3 | 30.4 | 14 | n.d. | n.d. | 0.3 | 2:1 |
| pMTL83251_P*_bgaL_*_C-FAST-AdhE2 induced | 1.6 | 0.05 | 37.0 | 13.3 | 0.1 | 0.4 | n.d. | 34 | 12.2 | 0.1 | 0.3 | n.d. | 3:1 |
| pMTL83251_P*_bgaL_*_C-FAST-AdhE2 non-induced | 1.8 | 0.05 | 35.3 | 14.9 | 0 | 0 | n.d. | 28.6 | 12.1 | 0 | 0 | n.d. | 2:1 |
| pMTL83251_P*_bgaL_*_N-FAST-AdhE2 induced | 1.7 | 0.06 | 43.1 | 14.3 | 0.2 | 0.6 | n.d. | 40.4 | 13.4 | 0.2 | 0.6 | n.d. | 3:1 |
| pMTL83251_P*_bgaL_*_N-FAST-AdhE2 non-induced | 2 | 0.06 | 35.3 | 14.9 | 0 | 0 | n.d. | 36.5 | 15.4 | 0 | 0 | n.d. | 2:1 |
| pMTL83251^3^ | 1.9 | 0.07 | 25.3 | 16 | 0 | 0 | 0 | 22.3 | 14.1 | 0 | 0 | 0 | 3:2 |
| pMTL83251_P*_thlA_*_C-FAST-Adc | 1.6 | 0.08 | 19.5 | 14.9 | 0 | 0 | 1.6 | 19.5 | 14.9 | 0 | 0 | 1.6 | 3:2 |
| pMTL83251_P*_thlA_*_N-FAST-Adc | 1.6 | 0.07 | 20.2 | 13.9 | 0 | 0 | 0.1 | 23.5 | 16.2 | 0 | 0 | 0.1 | 3:2 |

^1^ control strain during growth experiment with *E. limosum* [pMTL83251_P*_bgaL_*_AdhE2]

^2^ control strain during growth experiment with *E. limosum* [pMTL83251_P*_thlA_*_act]

^3^ control strain during growth experiment with *E. limosum* [pMTL83251_P*_thlA_*_C-FAST-Adc] and *E. limosum* [pMTL83251_P*_thlA_*_N-FAST-Adc]
